# Transcript Profile of the Response of Two Soybean Genotypes to Potassium Deficiency

**DOI:** 10.1371/journal.pone.0039856

**Published:** 2012-07-05

**Authors:** Cheng Wang, HaiFeng Chen, QingNan Hao, AiHua Sha, ZhiHui Shan, LiMiao Chen, Rong Zhou, HaiJian Zhi, XinAn Zhou

**Affiliations:** 1 National Center for Soybean Improvement, National Key Laboratory for Crop Genetics and Germplasm Enhancement, Nanjing Agricultural University, Nanjing, China; 2 Key Laboratory of Oil Crop Biology of the Ministry of Agriculture, Oil Crops Research Institute of the Chinese Academy of Agricultural Sciences, Wuhan, China; University of New England, Australia

## Abstract

The macronutrient potassium (K) is essential to plant growth and development. Crop yield potential is often affected by lack of soluble K. The molecular regulation mechanism of physiological and biochemical responses to K starvation in soybean roots and shoots is not fully understood. In the present study, two soybean varieties were subjected to low-K stress conditions: a low-K-tolerant variety (You06-71) and a low-K-sensitive variety (HengChun04-11). Eight libraries were generated for analysis: 2 genotypes ×2 tissues (roots and shoots) ×2 time periods [short term (0.5 to 12 h) and long term (3 to 12 d)]. RNA derived from the roots and shoots of these two varieties across two periods (short term and long term) were sequenced and the transcriptomes were compared using high-throughput tag-sequencing. To this end, a large number of clean tags (tags used for analysis after removal of dirty tags) corresponding to distinct tags (all types of clean tags) were identified in eight libraries (L1, You06-71-root short term; L2, HengChun04-11-root short term; L3, You06-71-shoot short term; L4, HengChun04-11-shoot short term; L5, You06-71-root long term; L6, HengChun04-11-root long term; L7, You06-71-shoot long term; L8, HengChun04-11-shoot long term). All clean tags were mapped to the available soybean (*Glycine max*) transcript database (http://www.soybase.org). Many genes showed substantial differences in expression across the libraries. In total, 5,440 transcripts involved in 118 KEGG pathways were either up- or down-regulated. Fifteen genes were randomly selected and their expression levels were confirmed using quantitative RT-PCR. Our results provide preliminary information on the molecular mechanism of potassium absorption and transport under low-K stress conditions in different soybean tissues.

## Introduction

Potassium (K) is one of the most abundant cations in plants. It is considered a vital soil resource. The fact that most plants can only absorb small amounts of soluble K from the soil frequently limits crop yield. Although concentrations of K^+^ in soil solution are in the range of only 0.1–6.0 mmol L^−1^, plants can still accumulate large amounts of this element, up to 2–6% of the total plant dry weight [1], [2]. K is widely distributed within plants and plays essential roles in maintaining plasma membrane potential, ion homeostasis, enzyme activation, signal transduction, and many other physiological processes in plant tissues [3]–[5]. For these reasons, inorganic K fertilizers are used on crops.

K^+^ obtained from the external environment is primarily absorbed by plant roots, which grow toward nutrient-rich areas and transport the nutrient to the root surface through the soil [6]. In addition to long-term K deprivation, plant roots can tolerate transient shortages of potassium from spatial and temporal variations in the availability of the nutrients. When plants are subjected to K^+^ deprivation, root cells sense the change in [K^+^]_ext_ and initiate a series of physiological reactions [7]. In recent years, the molecular mechanism of potassium uptake by plant roots and of the loading and transport within plants has become the focus of a great deal of research. Experimental data from various plants has demonstrated that there are both high- and low-affinity K^+^ transport systems [8], [9]. In comparative analyses, K^+^ absorption from soil to living plant cells and K^+^ transport inside plants are mediated by plant K^+^ transporters (high-affinity) and channels (low-affinity) [10]. Low-K stress conditions trigger the expression of high-affinity K^+^ transporters and up-regulate certain K^+^ channel genes in plants. Although the details of the molecular mechanisms related to the regulation of the sensing, signaling, and associated relevant gene activity in soybeans under low-K stress conditions are poorly understood, recent studies have provided some valuable clues. Over the past few decades, most molecular research into the absorption, loading/unloading, and transport of K in roots and leaves has used the model plant, *Arabidopsis thaliana*. For example, homology-based cloning has allowed the isolation of a large number of genes encoding K^+^ transporters and channels and the identification of their homologs in other plant species [11]–[13]. These transporters and channels make up the K^+^ absorption and transport system in plant cells. Seventy-one K^+^ transporter and channel genes have been isolated and identified in *Arabidopsis*. Some of these genes and their biological functions have been well described: several molecular and protein components have been shown to contribute to potassium uptake into plant roots. For example, AKT1 (shaker family) (*Arabidopsis thaliana* K^+^ transporter 1/2) encodes an inward-rectifying K^+^ channel. When identified in heterologous systems, AKT2 appears as a weakly-rectifying K^+^ channel that primarily expresses in the phloem of plant tissue [14], [15]. The K^+^ uptake permease (KUP) family is important for various physiological processes, and many of its members have been isolated and identified [16]. *Arabidopsis thaliana* high-affinity K^+^ transporter 1 (AtHKT1) has been shown to be a Na^+^/K^+^ transporter localized to the xylem parenchyma cells of leaves, mediating the Na^+^/K^+^ loading from xylem vessels into xylem parenchyma cells [17]. In addition to these transporters and channels, certain cytoplasmic enzymes in plant cells also have roles in K signal transduction, such as calcium signaling (CBL/CIPK) and transcription factors (REST) (repressor element-1 silencing transcription factor) [18], [19].

The genome of the soybean (*Glycine max*) has been completed sequenced. Schmutz *et al*. described the complete soybean genome sequence [20]. In that sequence a large number of genes were annotated using a variety of functional genomic methods. The availability of the genome sequences corresponding to these genes permit the characterization of their related functions. For example, the Solexa/Illumina platform permits comprehensive, accurate gene expression profiling [21]. There have been several recent studies of Solexa/Illumina sequencing technology in response to plant growth and development [22], [23].

In the present study, we used Solexa sequencing system to perform deep sequencing analysis, producing a great quantity of tags at a reasonable expense. We then used a digital gene expression (DGE) (digital gene expression) profile to filter the differentially expressed genes in different plant tissues [24]. In this way, rare transcripts were more likely to be identified with more reliable reproducibility (Pearson correlation coefficient with 0.8–0.95) [25]. The results of the Solexa sequencing technology tend to show strong correlations to those of quantitative PCR [26]. The objective of this study was to determine the expression levels of genes derived from low-K-tolerant and -sensitive varieties under low-K stress conditions to determine whether these genes had any effect on K utilization.

## Results

### Preliminary Screening of Soybean Varieties with Different Levels of Tolerance to Low-K Stress Conditions

To identify soybean varieties tolerant to low-K stress conditions, a total of 40 varieties (lines) were collected and subjected to preliminary screening under −K and +K conditions (K2 and K4, respectively) (Table S1). Three low-K-tolerant varieties (You06-71, XuDou8, and ZhongDou33) and two low-K-sensitive varieties (HengChun04-11 and ZhongHuang13) were identified in this preliminary screening. The difference between the low-K-tolerant varieties and low-K-sensitive varieties mainly involved potassium content, dry weight, accumulation of potassium, and potassium utilization efficiency (KUE) (potassium use efficiency), These indices were used to further screen and evaluate the varieties under the different K conditions.

### Effect of Different Levels of K on the Biomass of Plants of Different Genotypes

Biomass is the final result of plant growth and development. It reflects the extent to which the plant has suffered from the low-nutrition stress. It has always been a measure of resistance to the low-nutrition stress conditions. The experimental results indicated that different levels of K had a significant effect on the dry matter weights for different soybean varieties (Table 1). Under the low-K stress conditions, all five soybean varieties grew more poorly than under ordinary conditions but to different extents. The growth of low-K-tolerant varieties was better than low-K-sensitive varieties. You06-71 showed the highest coefficient of relatively dry weight (0.67), and HengChun04-11 showed the lowest coefficient of relatively dry weight (0.25). The trend among coefficients of relatively dry weight in different soybean varieties was as follows: You06-71 (0.67) > ZhongDou33 (0.53) > XuDou8 (0.51) > ZhongHuang13 (0.39) > HengChun04-11 (0.25). Plant roots are key to nutritional uptake under the conditions of both −K and +K. The dry weights of the root of low-K-tolerant variety You06-71 were 1.50 and 1.17 times of low-K-tolerant variety HengChun04-11 under the conditions of both −K and +K. This means that You06-71 has a stronger root system than HengChun04-11 and therefore stronger nutritional uptake capability. The shoot is the organ responsible for the transportation of nutrients and the photosynthetic product. The dry weights of the shoots of low-K-tolerant variety You06-71 were 3.86 and 1.19 times those of the low-K-sensitive variety HengChun04-11 under both −K and +K conditions. These results indicated that You06-71 caused more matter to accumulate. The ratio of the dry weights of shoots and roots of reflected the ability of the plant transport and distribute nutrition to the ground. You06-71 showed the highest ratio of shoot to root dry mass under the low-K stress conditions of any variety; this means that You06-71 can maintain more shoot growth while cannibalizing less of its root material. This may be one reason why it is so highly K efficient.

**Table 1:** Performance of five soybean genotypes under the low-K and normal-K conditions.

	Total dry weight (g/plant)			Ground biomass (g/plant)			Shoot/root	
Genotype	Low K	High K	Relative Index	Low K	High K	Relative Index	Low K	High K
You06-71	1.92a	2.85b	0.67	1.62a	2.29b	0.71	5.40	4.09
ZhongDou33	1.59b	2.98b	0.53	1.31b	2.46a	0.53	4.68	4.73
HengChun04-11	0.62c	2.46c	0.25	0.42c	1.98c	0.21	2.10	4.13
ZhongHang13	0.94c	2.38c	0.39	0.73c	1.93c	0.38	3.48	4.29
XuDou8	1.61b	3.18a	0.51	1.35b	2.57a	0.53	5.19	4.21

For each K level, means within a column followed by the different letter are significantly different according to the Duncan’s multiple range, P<5%, n = 5.

### Effect of Different Levels of K on the Relevant Indicators of K of Different Genotypes

**Figure 1:**
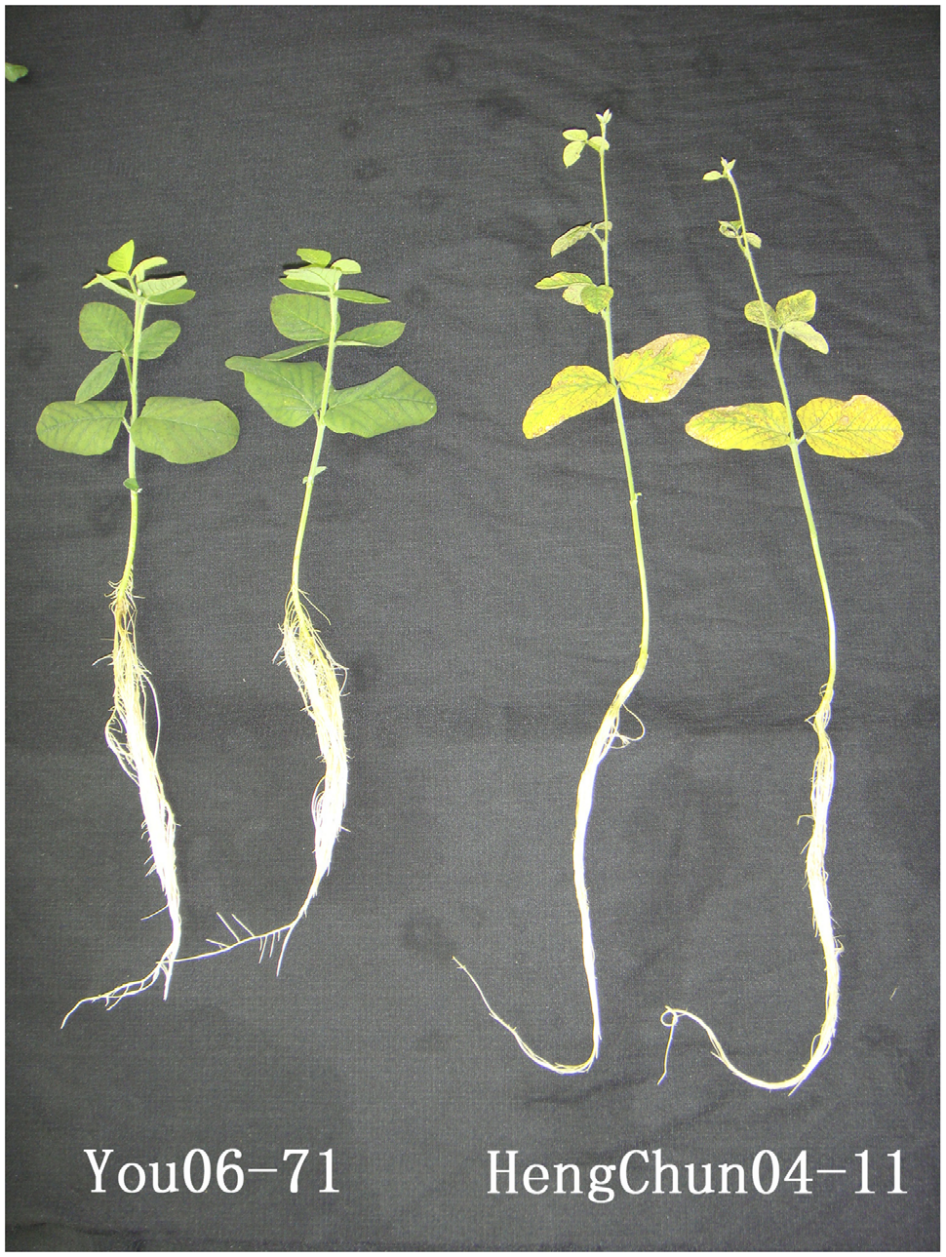
Comparison of the morphological changes of different soybean (Glycine max) varieties under low-K stress condition. You06-71 is the low-K tolerant variety; HengChun is the low-K sensitive variety.

Under low-K stress conditions, there were significant differences in the amount of K accumulated and the amount of KUE across different soybean varieties (Table 2), the accumulation of K is the product of dry weight and K content. The difference in dry weight is a major contributor to differences in K accumulation. The total K accumulation per plant of the low-K-tolerant You06-71 variety was 2.36 and 1.26 times of that of the low-K-sensitive variety HengChun04-11 under −K and +K conditions, respectively. In the shoots, K accumulation per You06-71 plant peaked under both −K and +K conditions. In addition, KUE in You06-71 plants were significantly higher under low-K stress conditions than +K conditions. These differences indicated that You06-71 had a pronounced ability to take up, distribute, and use K efficiently. In our experiment, under low-K stress conditions, the K content of HengChun04-11 was the highest out of all the soybean varieties studied, but it showed a weaker correlation to the plant’s total dry weight. The K content of You06-71 was the lowest out of all the soybean varieties studied, but showed the strongest correlation with total dry weight. This indicated that lower K content was sufficient to maintain the regular physiological functions of low-K-tolerant variety You06-71. The low-K-tolerant variety had two main characteristics, including strong ability to take up K and uses it efficiently. You06-71 showed an obvious advantage in the KUE and absorbed K more efficiently than other varieties, and differed from each other the least with respect to losses in total dry weight under low-K stress conditions. Among the five soybean varieties, HengChun04-11 showed an obvious disadvantage on these indices. Combined with the date of phenotype (Figure 1), we select You06-71 as the relatively low-K-tolerant variety and HengChun04-11 as the relatively low-K-sensitive variety to perform the following sequencing experiment.

**Table 2:** Potassium-related indicators in different genotypes under the low-K and normal-K conditions.

	Total K accumulation (mg/plant)			K accumulationin shoot (mg/plant)			Total K content (%)		KUE	
Genotype	Low K	High K	Relative Index	Low K	High K	Relative Index	Low K	High K	Low K	Relative Index
You06-71	26.86a	77.24a	0.35	16.94a	54.29a	0.31	1.40b	2.71a	137.24a	105.16b
ZhongDou33	22.82b	68.54b	0.33	12.35b	46.63b	0.26	1.44b	2.30b	110.78b	129.56a
HengChun04-11	11.38c	59.60b	0.19	8.94c	52.39a	0.17	1.84a	2.42b	33.79d	101.54b
ZhongHang13	13.35c	43.62c	0.31	9.49c	34.68c	0.27	1.42b	1.83c	66.19c	129.86a
XuDou8	22.45b	72.43a	0.30	15.78a	53.29a	0.30	1.39b	2.28b	115.46b	139.62a

For each K level, means within a column followed by the different letter are significantly different according to the Duncan’s multiple range, P<5%, n = 5.

### Solexa Sequencing Evaluation

In this study, to obtain a comprehensive view of the soybean gene expression profile under low-K stress conditions, we used the Solexa method to perform a high-throughput tag-sequencing analysis. Poly-(A)-enriched RNAs samples were prepared from You06-71(Y) and Hengchun04-11(HC) and used for low-K stress treatment lasting from 0.5 h to 12 d. The samples taken at 0.5, 2, 6, and 12 h were selected and used as the short-term library and those taken at 3, 6, 9, and 12 d were used as the long-term library. The following samples were used for Solexa sequencing: L1, You06-71-root short-term; L2, HengChun04-11-root short term; L3, You06-71-shoot short term; L4, HengChun04-11-shoot short term; L5, You06-71-root long term; L6, HengChun04-11-root long term; L7, You06-71-shoot long term; and L8, HengChun04-11-shoot long term. After sequencing, the number of tags for each library ranged from 5.50 to 5.99 million reads (raw tags) and the number of tags producing distinct sequences ranged from 0.3 to 0.5 million (Table 3). To determine whether the sequencing depth of the Solexa technology was sufficient for transcriptome coverage, the sequencing saturation was analyzed for all eight libraries. Saturation analysis confirmed whether the number of detected genes increased as the total number of tags increased. The number of detected genes plateaued when the number of sequences exceeded two million tags (10^6^) (Figure S1). This indicated that the size of the library was saturated and contained sufficient information for gene expression analyses and able to perform the next analysis.

**Table 3:** Categorization and abundance of tags.

Summary		L1	L2	L3	L4	L5	L6	L7	L8
Raw tags	Total	6249661	6082210	6025833	5777397	6170727	5889270	5940203	5909627
	Distinct tags	440455	574606	444939	426457	645685	602146	439324	363528
Clean tags	Total number	5985559	5704575	5741830	5503142	5736751	5496158	5667587	5683891
	Number ofdistinct tags	177778	199368	167498	158495	214218	210656	173094	143898
All tags mapping to gene	Total number	4743425	4433325	4755583	4582123	4536668	4447895	4788454	4767238
	Clean tags as% of total	79.25%	77.72%	82.82%	83.26%	79.08%	80.93%	84.49%	83.87%
	Number ofdistinct tags	96697	97305	91574	85365	101664	106076	91523	84341
	Clean tags as% of distinct	54.39%	48.81%	54.67%	53.86%	47.46%	50.36%	52.87%	58.61%
Unambiguous tagsmapping to gene	Total number	3650621	3425562	3442014	3331664	3455437	3266134	3315656	3443689
	Total % ofclean tags	60.99%	60.05%	59.95%	60.54%	60.23%	59.43%	58.50%	60.59%
	Number ofdistinct tags	76207	76010	72175	67777	79268	81635	71463	67372
	Clean tags as% of distinct	42.87%	38.13%	43.09%	42.76%	37.00%	38.75%	41.29%	46.82%
All Tag-mapped Genes	Number	33717	34993	34771	34291	35967	36353	35999	33739
	% of refgenes	50.92%	52.85%	52.52%	51.79%	54.32%	54.91%	54.37%	50.96%
Unambiguous tag-mapped genes	Number	26848	27443	27339	26889	28300	28540	28237	26752
	% of refgenes	40.55%	41.45%	41.29%	40.61%	42.74%	43.11%	42.65%	40.40%
Mapping to mitochondrion	Total number	201	297	161	191	267	319	216	140
	Clean tags as% of total	0.00%	0.01%	0.00%	0.00%	0.00%	0.01%	0.00%	0.00%
	Number ofdistinct tags	25	33	29	22	39	33	32	16
	Clean tags as% of distinct	0.01%	0.02%	0.02%	0.01%	0.02%	0.02%	0.02%	0.01%
Mapping to chloroplast	Total number	4185	6981	29820	22891	8466	9592	36984	12778
	Clean tags as% of total	0.07%	0.12%	0.52%	0.42%	0.15%	0.17%	0.65%	0.22%
	Number ofdistinct tags	244	341	481	422	386	360	487	362
	Clean tags as% of distinct	0.14%	0.17%	0.29%	0.27%	0.18%	0.17%	0.28%	0.25%
Mapping to genome	Total number	633969	598340	570702	564597	613028	518917	531827	578709
	Clean tags as% of total	10.59%	10.49%	9.94%	10.26%	10.69%	9.44%	9.38%	10.18%
	Number ofdistinct tags	38961	47412	46550	45832	55250	47841	51542	36698
	Clean tags as% of distinct	21.92%	23.78%	27.79%	28.92%	25.79%	22.71%	29.78%	25.50%
Unknown tags	Total number	603779	665632	385564	333340	578322	519435	310106	325026
	Clean tags as% of total	10.09%	11.67%	6.72%	6.06%	10.08%	9.45%	5.47%	5.72%
	Number ofdistinct tags	41851	54277	28864	26854	56879	56346	29510	22481
	Clean tags as% of distinct	23.54%	27.22%	17.23%	16.94%	26.55%	26.75%	17.05%	15.62%

Clean tags are those remaining after low- quality tags have been removed from the raw data. Distinct tags are different types of clean tags. Unambiguous tags are the clean tags remaining after removal of tags mapped to reference sequences from multiple genes. The number of the total clean tags and distinct tags from the eight libraries that matched to the mitochondrion, chloroplast, and genome sequences were listed.

Note: L1, You06-71-root short term; L2, HengChun04-11-root short term; L3, You06-71-shoot short term; L4, HengChun04-11-shoot short term; L5, You06-71-root long term; L6, HengChun04-11-root long term; L7, You06-71-shoot long term; L8, HengChun04-11-shoot long term.

**Figure 2:**
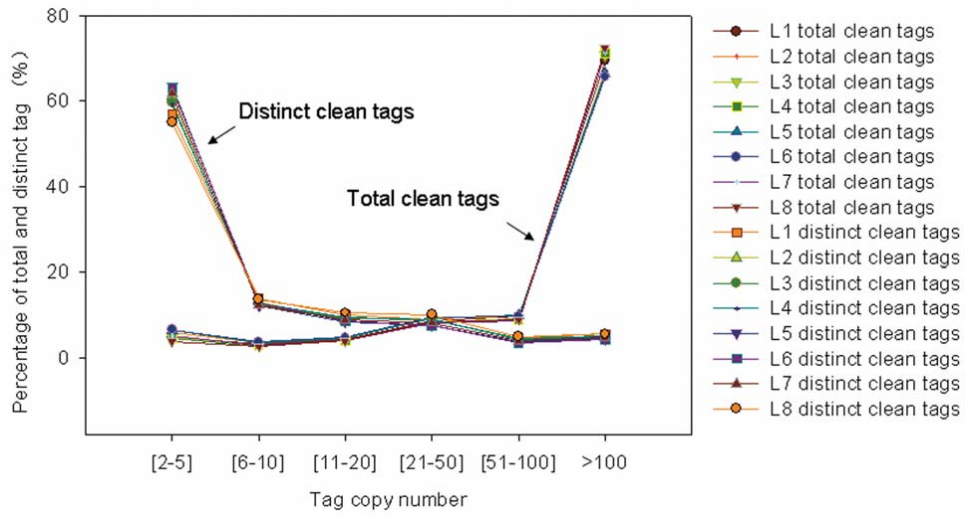
Distribution of total clean tag and distinct tag counts over different tag abundance categories from eight libraries. The x-axes represent percentage of total clean and distinct tag (%). The y-axes represent the tag copy number. Clean tags (tags used for analysis after removal of dirty tags), distinct tags (all types of clean tags). L1, You06-71-root short term; L2, HengChun04-11-root short term; L3, You06-71-shoot short term; L4, HengChun04-11-shoot short term; L5, You06-71-root long term; L6, HengChun04-11-root long term; L7, You06-71-shoot long term; L8, HengChun04-11-shoot long term.

After filtering dirty tags from raw data, a total of 5,985,559; 5,704,575; 5,741,830; 5,503,142; 5,736,751; 5,496,158; 5,667,587; and 5,683,891 clean tags were found to correspond to 177,778; 199,368; 167,498; 158,495; 214,218; 210,656; 173,094; and 143,898 distinct tags for the L1, L2, L3, L4, L5, L6, L7, and L8 libraries (Table 3). The distribution of the various tag abundance categories between total and distinct tag counts showed different results for all libraries. These distributions can be used to evaluate the normality of the whole data (Figure 2). Among the total clean tags, more than 5% had 2–5 copies (5.28% on average), 25.48% of the tags had 5–100 copies, and more than 65% of the tags had more than 100 copies (69.24% on average). Among the distinct clean tags, more than 60% had 2–5 copies (60.01% on average), 34.31% of the tags had 5–100 copies, and fewer than 5% of the tags had more than 100 copies (4.95% on average).

Matching reads to genes is a critical step in the prediction of sequences and determination of the molecular events underlying gene expression. All clean tags were BLAST searched against the available genome (http://phytozome.net/). Then 66210 genes were used for the initial analysis, and 61,044 of these genes (92.2%) had CATG sites. Out of the eight data sets representing expressed sequences and transcriptomes from the eight libraries, an average of 81.43% of all sequence were matched by tags and an average of 15.48% of distinct tags were uniquely mappable to the reference sequences. The tags mapping to the database generated 26,848; 27,443; 27,339; 26,889; 28,300; 28,540; 28,237; and 26,752 tag-mapped transcripts for the L1, L2, L3, L4, L5, L6, L7, and L8 libraries, respectively. However, due to the presence of incomplete sequences, a certain number of ambiguous tags (an average of 58.66%) could not be aligned to reference genes.

In addition, sense and antisense genes play important roles in gene expression and regulation, sequencing tags mapping to the complementary strands of the sense gene would suggest that the antisense strand also had transcripts [27]. In our study, approximately 23.30% of the tags in the eight libraries mapped to the antisense strands (Table S2), indicating that those genes might be activated via sense or antisense regulation.

### Functional Classification of Differentially Expressed Transcripts

GO (gene ontology) enrichment analysis of functional significance was used to evaluate the potential function of genes that showed significant differences between the two soybean varieties under low-K stress conditions. Gene ontology (GO) is an internationally standardized gene function classification system for comprehensively describing the properties of genes and their products in any organism. GO has three ontologies: molecular functions, cellular components, and biological processes. The basic unit of GO is the GO-term. Every GO-term belongs to a type of ontology. In gene expression profiling analysis, GO enrichment analyses of functional significance was performed using hypergeometric testing to map all differentially expressed genes to terms in the GO database (http://www.geneontology.org/) by looking for GO terms that were significantly enriched in a given DEG (differentially expressed gene) relative to the genome background.

**Figure 3:**
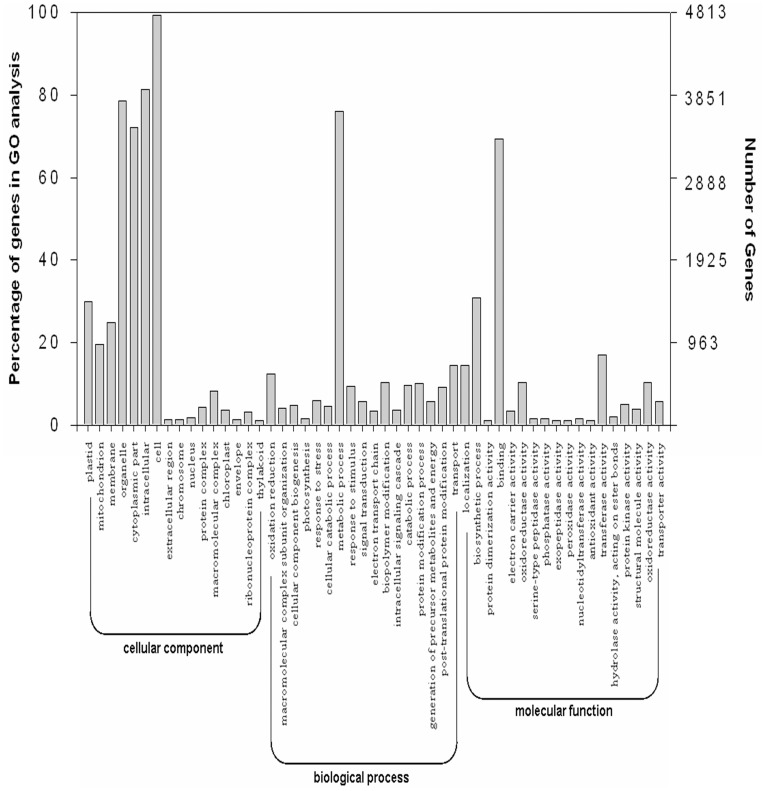
Histogram presentation of gene ontology classification. It includes three main categories: biological processes, cellular components, and molecular functions. The y-axes on the left and right indicate the percentage of a specific category of genes in that main category and the number of genes in a category, respectively.

In our study, predicted and known transcripts were divided into groups: molecular function transcripts, cellular component transcripts, and biological process transcripts. Of these, 4813 differentially expressed transcripts were divided into 50 functional groups (Figure 3). For molecular function, the most significant enrichment was observed among the various binding genes (GO: 0005488), including 40 DEGs encoding calcium ion binding proteins (GO: 0005509), 66 DEGs encoding iron ion binding proteins (GO: 0005506), 55 DEGs encoding zinc ion binding proteins (GO: 0008270), and 4 DEGs encoding potassium ion binding proteins (GO: 0030955). Two hundred and eighty DEGs were found to encode transferase activity (GO: 0016740), these included kinase activity genes (GO: 0016301). Ninety-four DEGs were found to encode transporter activity (GO: 0005215). These included transmembrane transporter activity genes (GO: 0022857). For cellular components, 99.29% of DEGs were found to be involved in the “cell” structure. These included genes involved in the plasma membrane and external encapsulating structures such as the cell wall and cell envelope. There were 524 DEGs encoded in membranes (GO: 0016020). These included plastid membranes (GO: 0005886) and mitochondrial membranes (GO: 0031966). For biological processes, 813 DEGs were found to be involved in metabolic process (GO: 0008152) and 62 DEGs were found to be involved in stress response (GO: 0006950), including responses to salt stress (GO: 0009651), osmotic stress (GO: 0006970), ethylene stimulus (GO: 0009723), and oxidative stress (GO: 0034599).

**Figure 4:**
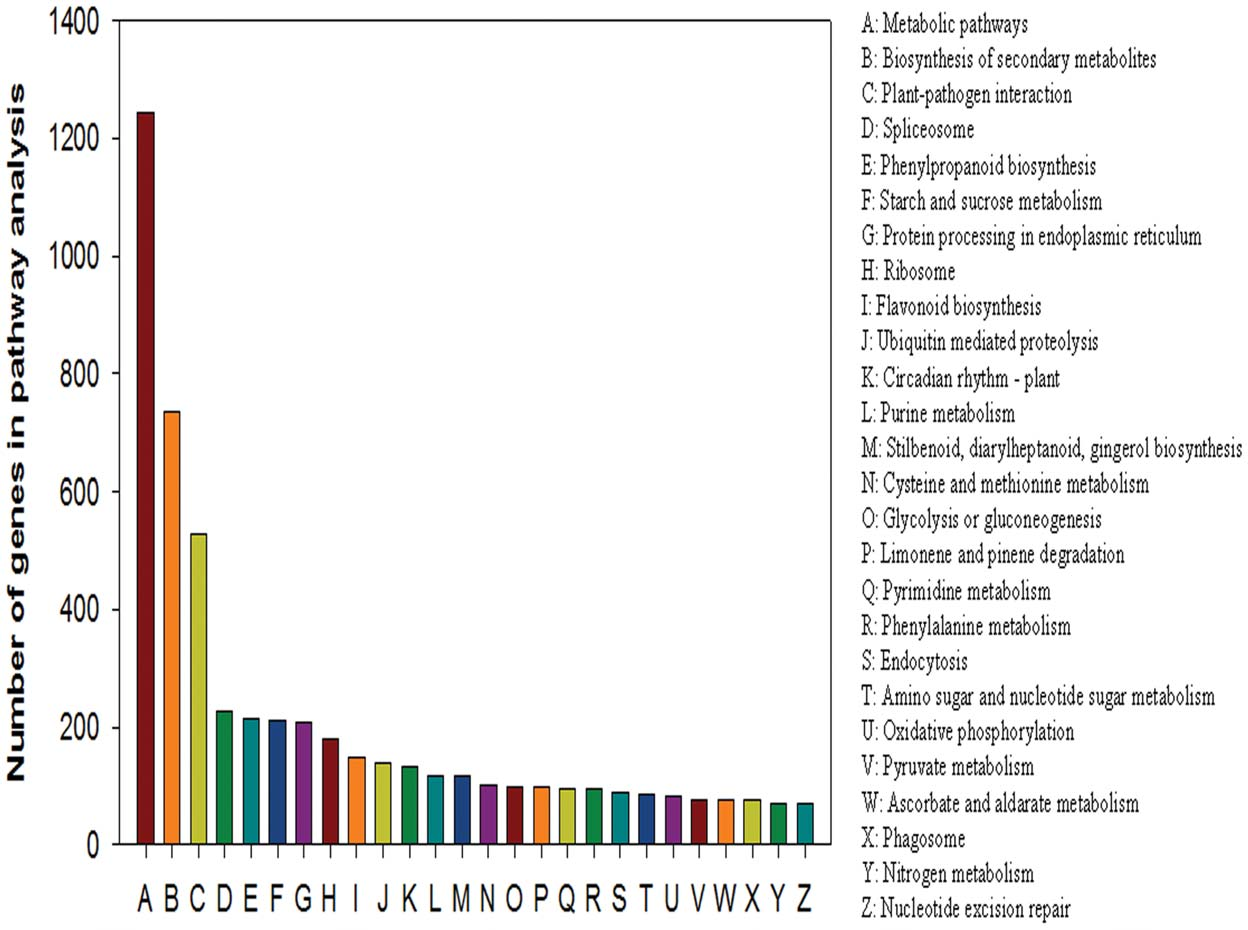
Enrichment analysis of the 26 (A-Z) main pathways. The x-axes represent the category of each pathway. The y-axes represent the number of genes in pathway analysis.

We also matched differentially expressed genes to terms in the Kyoto Encyclopedia of Genes and Genomes (KEGG) database. KEGG is the major public pathway-related database. Different genes usually cooperate with each other to exercise their biological functions. Pathway-based analysis helps the user to further understand the biological functions of specific genes. Pathway enrichment analysis identifies significantly enriched metabolic pathways and signal transduction pathways in DEGs by comparing them to the whole-genome background [28]. The formula used to determine DEGs is the same as that used in GO analysis. We arranged 5440 differentially expressed genes to 118 KEGG pathways (Figure 4). The pathways with the greatest numbers of unique sequences were all metabolic pathways (1435 members) (ko01100). Metabolic pathways are large complexes comprising several networks. These networks can involve biosynthesis of secondary metabolites, carbohydrate metabolism, lipid metabolism, or amino acid metabolism [29]–[32]. Other pathways, such as flavonoid biosynthesis (150 members) (ko00941), purine metabolism (118 members) (ko00230), ABC transporters (44 members) (ko02010), and ubiquitin mediated proteolysis (140 members) (ko04120) were also involved (Table S3) [33]–[36]. We believe that these pathways play a significant role in the plant response to low-K-stress conditions.

### Genes Found to be Differentially Expressed in the Two Soybean Varieties

**Figure 5:**
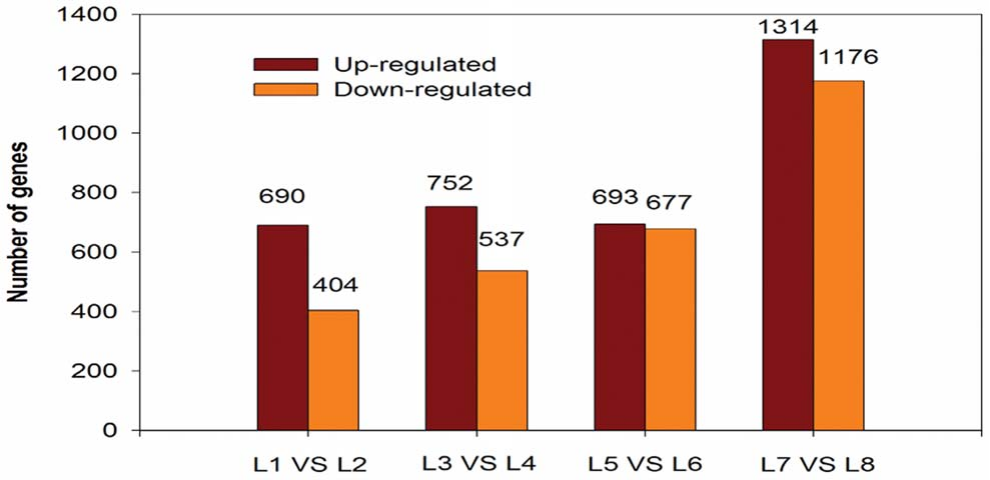
Number of up-regulated genes and down-regulated genes with significant differential expression among different sample libraries. L1, You06-71-root short term; L2, HengChun04-11-root short term; L3, You06-71-shoot short term; L4, HengChun04-11-shoot short term; L5, You06-71-root long term; L6, HengChun04-11-root long term; L7, You06-71-shoot long term; L8, HengChun04-11-shoot long term.

Schmid *et al*., Ligeng Ma *et al*., and Aceituno *et al*. concluded that the *A. thaliana* transcriptome varied strongly from one organ to another, and showed organ-specific expression during development [37]–[39]. These studies suggest that specific plant organs had their own independent transcriptomes. In soybeans, by comparing our eight Solexa libraries, we identified a large number of differentially expressed transcripts. The existences of differentially expressed genes in plants hint at the molecular basis of root and shoot development under low-K stress conditions. With reference to the significance of digital gene expression profiles, we adopted a rigorous algorithm to identify differentially expressed genes in their two corresponding libraries (eg: L1 vs L2) (regulated genes were those with a |log2 ratio| >1, FDR <0.001, *P*<0.01) [24]. Analysis of the eight libraries revealed 24,554; 24,831; 24,767; 24,325; 25,593; 25,859; 25,384; and 24,544, tag-mapped sense transcripts for L1, L2, L3, L4, L5, L6, L7, and L8. We found that many genes were differentially expressed in the two libraries (comparing the transcripts originating from low-K-tolerant and low-K-sensitive plants). The results showed 13,231; 13,963; 15,091; and 17,285 differentially expressed genes for L1 vs. L2, L3 vs. L4, L5 vs. L6, and L7 vs. L8, respectively. After filtering with FDR<0.001, *P*<0.01 and |log2Ratio| >1 and excluding genes with unknown function (average 41.89%) (Table S4), a total of 1094 differentially expressed genes were detected in L1 vs. L2. These included both up-regulated genes (690) and down-regulated genes (404). A total of 1289 differentially expressed genes were detected in L3 vs. L4. These included both up-regulated genes (752) and down-regulated genes (537). A total of 1370 differentially expressed genes were detected in L5 vs. L6. These included both up-regulated genes (693) and down-regulated genes (677). A total of 2490 differentially expressed genes were detected in L7 vs. L8. These included both up-regulated genes (1314) and down-regulated genes (1176) (Table S5) (Figure 5). The predicted genes in different libraries are shown in Table S6.

**Figure 6:**
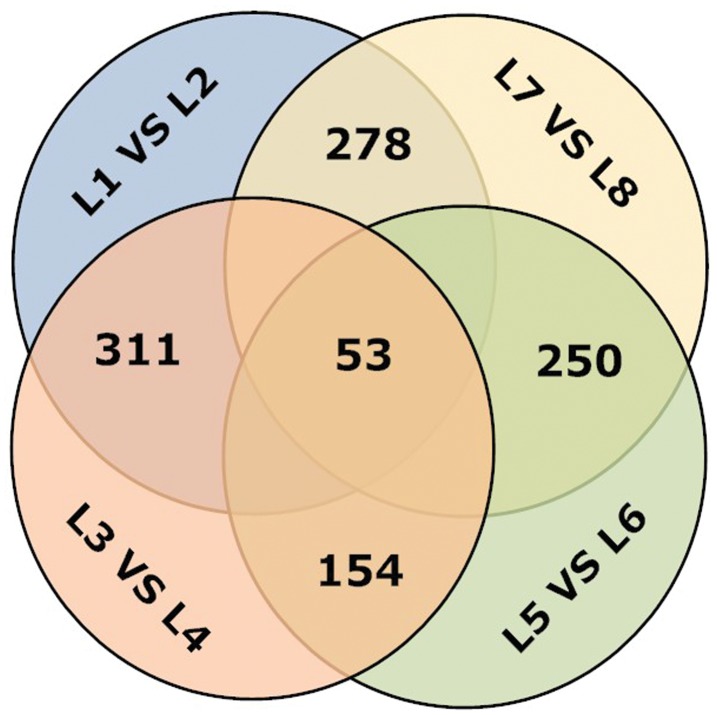
Analysis of tag-mapped transcripts (genes) in eight libraries. L1, You06-71-root short term; L2, HengChun04-11-root short term; L3, You06-71-shoot short term; L4, HengChun04-11-shoot short term; L5, You06-71-root long term; L6, HengChun04-11-root long term; L7, You06-71-shoot long term; L8, HengChun04-11-shoot long term.

In addition, in data sets from roots and shoots, a large number of genes showed differential expression between short term (0–12 h) and long term (3–12 d). This suggests that many genes were regulated in growing plants under low-K stress conditions. The DGE results revealed 279 genes that were differentially expressed in both the short term and long term in roots and 532 genes that were differentially expressed in the both short term and long term in shoots. There were 53 genes that were continuously expressed in all data sets (Table S7, Figure 6). Meanwhile, we presented the 10 most differentially expressed genes in the L1/L2, L3/L4, L5/L6, L7/L8 libraries, for a total of 40. The relative abundance is expressed as a TPM (number of transcripts per million clean tags; when one tag matched one gene perfectly. TPM represented the level of specific gene expression) ratio of the target group to that of the control group. As shown in Table 4, these genes included transcription factors, protein kinases, and other gene products.

**Table 4:** The most differentially expressed annotated genes in L1 vs. L2, L3 vs. L4, L5 vs. L6, and L7 vs. L8 libraries based on expressed tag frequency (TPM: transcripts per million).

Gene ID	TPM ratio	Functional annotation	Gene ID	TPM ratio	Functional annotation
L1 VS L2 up			L5 VS L6 up		
Glyma08g28800	6900	Full = 60S ribosomal protein L30	Glyma03g33620	1738	ADP-glucose small subunit PvAGPS1
Glyma19g38570	3859	Syntaxin, plant, putative	Glyma18g09290	1160	Disease resistance protein
Glyma02g03230	2924	Matrix metalloproteinase	Glyma13g23060	1325	Sucrose-phosphate synthase
Glyma13g23060	1119	Sucrose-phosphate synthase	Glyma18g51930	1238	NBS-LRR disease-resistance protein
Glyma14g09540	986	MYB transcription factor MYB81	Glyma20g27410	697	Receptor-like kinase homolog RK20-1
L1 VS L2 down			L5 VS L6 down		
Glyma16g30630	2384	Leucine-rich repeat	Glyma09g30360	418	Purple acid phosphatase
Glyma12g18190	1946	TNP1	Glyma06g37150	309	S-adenosylmethionine decarboxylase
Glyma07g07100	1508	Rpp4 candidate 3	Glyma11g13110	273	F-box and wd40 domain protein
Glyma09g38550	1052	Pyrophosphate isomerase	Glyma18g09290	237	Cytochrome P450 enzyme
Glyma13g25800	701	Protein tyrosine kinase	Glyma18g50690	17.15	Serine-threonine kinase
L3 VS L4 up			L7 VS L8 up		
Glyma08g28800	2996	Full 60S ribosomal protein L30	Glyma02g47460	8787	ADP-glucose small subunit PvAGPS1
Glyma04g40720	1498	60S acidic ribosomal protein P1	Glyma19g38570	5752	Syntaxin, plant, putative
Glyma10g02690	940	FIE	Glyma02g03230	5364	Matrix metalloproteinase
Glyma19g05370	819	Cell division protease	Glyma12g01260	2982	Serine carboxypeptidase
Glyma02g03420	801	UDP-glucosyltransferase	Glyma16g33910	1994	R 8 protein
L3 VS L4 down			L7 VS L8 down		
Glyma09g31740	927	Dehydrin-like protein	Glyma07g07100	5542	Rpp4 candidate 3
Glyma18g04520	672	High mobility group family	Glyma12g33150	1196	Structural constituent of cell wall
Glyma12g11390	509	MYB transcription factor MYB117	Glyma16g06070	1108	Ribonucleoside-diphosphate reductase chain
Glyma10g07770	491	Dehydration-responsive protein	Glyma06g35630	1003	Tyrosine aminotransferase
Glyma13g07530	418	Ubiquitin-protein ligase	Glyma01g41280	968	Polyprotein

### Differentially Expressed Genes Encoding Transcription Factors

Transcription factors are involved in the regulation of gene expression in response to stress in higher plants [40]. Changes in gene transcription are associated with changes in the expression of transcription factors. Our DEG results showed that 144 genes encoding transcription factors were induced 2.02 to 968-fold, including 77 up-regulated and 67 down-regulated genes. Among the 144 genes, 47 were from the MYB gene family, 15 were bZIP (basic region/leucine zipper motif) transcription factors, 8 were AP2-EREBP transcription factors, 7 were GRAS (GAI, RGA, SCR domain transcriptional regulators) family transcription factors, 6 were WRKY transcription factors, 6 were heat shock transcription factors, 4 were CPP (cystein-rich polycomb-like protein) transcription factors, 3 were ethylene-responsive transcription factors, 3 were ERF domain-containing transcription factors, 2 were bHLH transcription factors, 2 were AN1-like transcription factors, 1 was a BEL1-like transcription factor, 1 was a GATA transcription factor, and one was a TGA10 transcription factor [41]–[47]. The others were mainly putative transcription factors (Table S8).

### Differentially Expressed Genes Encoding Kinases

Kinases play important roles in the development of eukaryotic cells. They operate in a large number of distinct perception and signaling pathways, such as the signal transduction pathways controlling cell growth, differentiation, and death [48]. The response of cells to stress is mediated by a large number of protein kinases [49]. Protein kinases also act as molecular switches to regulate potassium channels [50]. Among our DEGs, a total of 248 genes encoding kinases were identified, including 147 up-regulated and 101 down-regulated genes. Among these genes, 29 were serine or threonine protein kinases, 13 were calcium-dependent protein kinases (CDPKs), 6 were CBL-interacting protein kinases, 5 were tyrosine kinases, 4 were leucine-rich repeat transmembrane protein kinases, 3 were wall-associated kinases, 2 were MAP-kinases, 1 was a stress-induced receptor-like kinase, and 1 was a kinase-associated protein phosphatase [51]. Another 40 were different types of receptor-like kinases, and the remaining genes coded for other types of kinases (Table S9).

### Potassium Absorption- and Transportation-related Genes

Potassium absorption and transportation are essential physiological and biochemical processes for plants. Among our DEGs, a total of 12 genes encoding potassium channels and ion transporters showed significant differential expression in this study, including eight up-regulated genes (Glyma13g23960, Glyma07g30360, Glyma08g09720, Glyma12g29190, Glyma07g08030, Glyma15g10140, Glyma08g20030, and Glyma08g39840), three down-regulated genes (Glyma08g01900, Glyma10g24620 and Glyma05g33660), and one bidirectionally regulated genes (Glyma03g31130).

### Oxidative Stress-related Genes

Reactive oxygen species (ROS) are known to be involved in signaling pathways specific to low-K stress potassium conditions [52]. Peroxidase, cytochrome P450, and glutathione S-transferase play important roles during oxidative stress in plants [53]. In our study, a total of 70 genes encoding oxidative stress-related products were differentially expressed under low-K stress conditions. These included 39 up-regulated and 31 down-regulated genes. Among these, 34 cytochrome P450 genes and 19 lipoxygenase genes were identified. Genes encoding peroxidase and glutathione S-transferase were not found among the DEGs (Table S10).

### Genes Involved in Carbohydrate and Energy Metabolism

A large number of differentially expressed genes were found to be involved in carbohydrate and energy metabolism. These included glycolysis and the citrate cycle (TCA cycle), oxidative phosphorylation, photosynthesis, and nitrogen metabolism. Glycolysis metabolism is the process of converting glucose into pyruvate and generating small amounts of ATP (energy) and NADH (reducing power). This process has been reported to play an important role in potassium nutrition in plants [54]. Among our DEGs, 17 genes involved in glycolysis showed increased transcript abundance. These genes encoded, among other products, ADP-glucose pyrophosphorylase (Glyma02g47460; 8,787-fold), sucrose-phosphate-synthase (Glyma13g23060; 1,325-fold), and UDP-glucose glucosyltransferase (Glyma11g00230; 3.24-fold). Glyma02g47460 (8,787-fold) showed the highest TPM up-regulation ratio. Phosphorylation is the metabolic pathway that indirectly regulates the ATP-sensitive potassium channels [55]. Out of all the genes involved in phosphorylation, 73 showed increased transcript abundance. These included the genes encoding kinase-associated protein phosphatase (Glyma11g00630-11.61-fold), tyrosine phosphatase (Glyma20g31390-2.92-fold), and chloroplast pentose phosphate transporter (Glyma10g11430-2.1-fold). Photosynthesis is the process by which green plants use light energy to synthesize organic compounds from carbon dioxide and water. Twelve genes involved in photosynthesis were differentially expressed. These included one down-regulated chlorophyll a/b binding protein gene (Glyma16g27990-2.15-fold) and two up-regulated photosynthetic reaction center protein genes (Glyma05g00620-3.32-fold, Glyma20g28300-5.10-fold).

### Other Differentially Regulated Genes

One other type of high-level differentially expressed gene related to low-K stress conditions was shown. After analysis of the DEGs, five genes encoding the ATP binding cassette (ABC) transporter family were found to be differentially expressed (Glyma09g04980, Glyma05g32620, Glyma08g00280, Glyma08g17110, and Glyma08g10710). Four defense response genes were also identified, those encoding abscisic stress response protein (Glyma20g30720), NBS-LRR type disease resistance proteins (Glyma12g36510), wound-induced basic proteins (Glyma08g46240), and salt-tolerance protein (Glyma12g05570). In addition, 16 genes related to oxidoreductase activity were identified, those encoding alcohol dehydrogenase (Glyma08g39520), NADPH-specific isocitrate dehydrogenase (Glyma14g39160), a short-chain dehydrogenase (Glyma16g04630), fatty acid desaturase (Glyma01g29630), and others. In addition, two gibberellin-responsive protein genes were found to be up-regulated under low-K stress conditions (Glyma17g36220-4.11-fold, Glyma06g12700-2.34-fold). One gene encoding a BURP domain protein (Glyma14g20440) and ten encoding dehydration-responsive binding proteins were differentially expressed. The expression of Glyma09g08330 and Glyma15g19910 was found to be up-regulated. The others were down-regulated.

### Quantitative Real-time PCR (qRT-PCR) Confirmation

**Figure 7:**
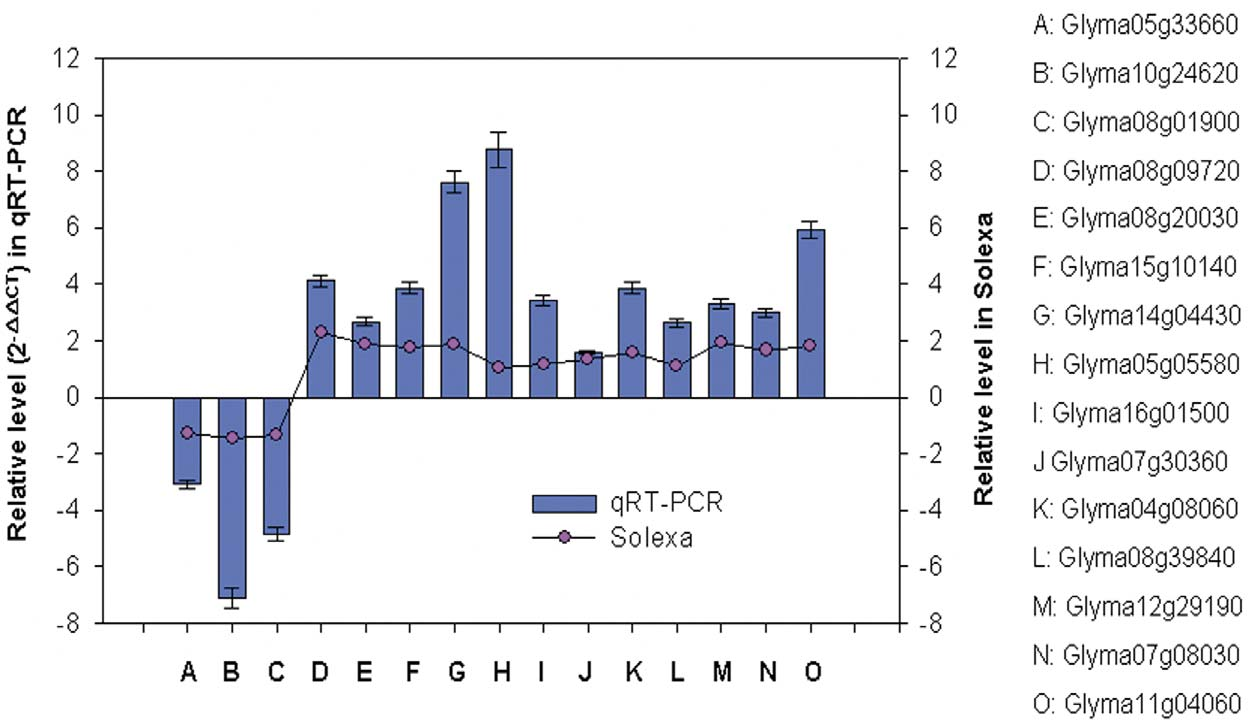
Validation of the Solexa results using qRT-PCR method. The y-axes on the left and right indicate the relative expressed level of tag-mapped genes in qRT-PCR and in Solexa sequencing analysis, respectively. The expression level of fifteen selected genes was measured by qRT-PCR. TUA5, UBQ10 and UKN2 were chosen as an endogenous reference.

To confirm the reliability of Solexa/Illumina sequencing technology, 15 candidate genes were confirmed by qRT-PCR using RT-primers (Table S11). We used the geNorm software to evaluate the stable of housekeeping genes.TUA5 and UBQ10 were ranked the most stable of all samples. UKN2, ACT2/7 and HDC consistently ranked poorly. The optimal numbers of housekeeping genes required for RT-PCR date normalization were determined by the geNorm (Table S12). Finally, three housekeeping genes (TUA5, UBQ10, and UKN2) were selected to normalize the level of gene expression (V3/4 = 0.133<0.15) (Table S13). Differential expression was observed for all the candidate genes, indicating that they are involved in the regulatory networks that are active during low-K stress condition. The results of RT-PCR showed that the qRT-PCR assessments (relative expressed level) of these 15 genes (tag-mapped genes) were consistent with those of Solexa sequencing analysis (Figure 7).

## Discussion

Soybeans require a great deal of soluble potassium. The mechanism of high K use efficiency of plant was complicated; it can be affected by different factors, such as plant’s ability to take up, transport, distribute, and utilize K. Therefore, screening the soybean germplasm for highly K-efficient varieties and varieties with improved of K-nutritional traits may be an effective way to relieve the plant’s demand for potassium. In our experiment, the growth of five soybean varieties with different low-K tolerance were reduced to different degrees as the concentration of K decreased. Low-K-sensitive varieties were affected more than the low-K-tolerant varieties. You06-71 has a high dry weight, high potassium accumulation, high KUE, and low potassium content under the low-K stress conditions, but the variety HengChun04-11 does not. These results indicate that variety You06-71 has a greater capability to take up K and use it efficiently than HengChun04-11. The selection of a suitable variety was critical to the following sequencing experiment.

The objective of the present study was to perform a preliminary exploration of transcripts involved in the development of soybean roots and shoots under low-K stress conditions and to provide background work for investigations of the mechanism behind their regulation. There are few existing reports showing the transcriptional changes associated with the absorption and transportation of potassium in soybean root and shoot transcript profiles under low-K stress conditions, and those that are available do not include deep sequencing results. For this reason, we used the Solexa system to analyze differential transcript abundance and regulations in response to low-K stress conditions in two soybean varieties (You06-71 and HengChun04-11). In our experiments, the differentially expressed genes were screened using transcriptome annotation. We discovered that several were involved in the regulation of the absorption and transportation of potassium and of metabolic pathways. Several other biological processes that had not previously been linked to K stress, such as natural killer-cell-mediated cytotoxicity, thiamine metabolism, monoterpenoid biosynthesis, zeatin biosynthesis, the phosphatidylinositol signaling system, and the biosynthesis of flavone and flavonol, were dramatically altered during K-stress [56]–[59]. These may be novel genes relevant to potassium use efficiency (KUE) in soybeans.

In the present study, many potential potassium-regulated genes were identified in roots and shoots. In plants, potassium processing involves several steps, including uptake, translocation, and recycling [60], [61]. Some of the candidate genes identified in this study may play important roles in these processes. These include putative potassium transporters, potassium channel, and potassium channel regulatory factors. The K^+^ uptake permeases (KUPs) encode plasma membrane K^+^/H^+^ symporters [62]. They have been found in plasma membranes of various cell types and are thought to catalyze K^+^ influx in to cells under low-apoplastic-K^+^ concentrations [63]. The transcript representing the KUP related potassium transporter was Glyma08g09720, which showed up-regulated expression (4.95-fold) in the low-K-tolerant variety, implying that this gene might assist the KUP gene family in K^+^ absorption under low-K stress conditions. AKT2 encoded voltage-independent K^+^-channels expressed in the phloem and xylem parenchyma. It has been implicated in both the loading and unloading of the phloem and is responsible for the recirculation of K^+^ from the shoots to the roots [64]. A potassium channel transcript (Glyma08g20030) that was determined by a sequence analysis to represent the AKT2 gene was hypothesized to be linked to the ion transport process. AtKC1 is a shaker-like channel family gene. It has been identified as an important, dual-affinity, inward-rectifying K^+^ channel that participates in K^+^ uptake from soil into cells in *Arabidopsis* roots [65]. Recent studies using electrophysiological techniques demonstrated that AtKC1 could not form a functional K^+^ channel by itself but that it might form one with AKT1. The inhibition of AKT1-mediated inward K^+^ currents was significantly enhanced by the injection of AtKC1 cRNA [66]. In our study, the transcript representing the AtKC1 gene (Glyma05g33660) was down-regulated in the low-K-tolerant variety. In the short-term, the expression level of this gene was higher in HengChun04-11 than in You06-71. This might explain why soybean You06-71 is tolerant to low-K conditions and soybean HengChun04-11 is not. KAT1 is a highly selective inward-rectifying potassium channel. This voltage-gated channel mediates long-term potassium influx into guard cells, leading to stomatal openings [67]. Expression of the transcript representing the KAT1 gene, Glyma15g10140, was found to be up-regulated (1.63-fold, 3.39-fold) in the low-K-tolerant variety during both periods, suggesting that this variety was better able to perceive potassium deficiency, open the stoma, and stimulate potassium transportation. Another putative potassium transporter may play a key role in K assimilation and translocation and could improve stress tolerance in soybeans.

We also noticed a significant phenomenon involving several transcription factors. A single transcription factor can regulate the expression of many genes. Transcriptional control of the expression of stress-response genes is a crucial part of the plant response to a range of abiotic and biotic stresses [68]. This means that transcription factors can show some complexity and overlap in their responses to different stressors and are likely to lead to new ways of enhancing crop tolerance to environmental stress [69]. Some transcription factors are related to the development of potassium-related metabolic pathways [70]. For example, the expression level of the Glyma04g08060 (WRKY50) (2.97-fold) gene was up-regulated in the low-K-tolerant variety over the short term. WRKY50 is a defense-related transcription factor belonging to the WRKY family of proteins [46]. Armengaud *et al*. pointed out that increased jasmonic acid (JA) levels in K^+^-starved plants might repress otherwise inducible defense responses [71]. Although WRKY50 proteins have not been reported to regulate the expression of JA signaling components, the absence of these proteins relieves this repression and restores JA-derived signaling in plants [72]. In this way, WRKY50 may have positive effects under low-K stress conditions. In our study, several AP2/EREBP (apetala 2-ethylene-responsive element binding proteins) transcription factors were found to be among our DEG. The AP2/EREBP family proteins are unique to plants and share a highly conserved region of about 60 to 70 amino acids [44]. Many AP2/EREBP transcription factors can activate genes in response to abiotic stress and are involved in the ABA response [43], [73]. The expression levels of these genes were up-regulated in the roots and shoots of the low-K-sensitive plant varieties. We assume that these genes can indirectly regulate the potassium channel involved in K-metabolic pathways in soybeans. The function of this gene will be studied by RNA-interference or by over-expression in transgenic plants in the future. In addition, we found many members of the MYB transcription factor family to be differentially expressed between two varieties (i.e., Glyma11g22960, Glyma07g05960, etc.). Some reports have suggested that these genes regulate a wide array of processes in plants, including the synthesis of secondary metabolites, different aspects of plant development, abiotic stress responses, and other processes [41]. This indicates that the soybean MYB family genes may be responsive to K-stress. One gene encoding GATA transcription factors (Glyma11g04060) was found to be up-regulated (3.51-fold) in both soybean varieties. Many GATA factors can activate or inactivate genes in response to environmental deficiencies [74]. For this reason, we assume that this gene is involved in low K- resistance in soybeans.

We also found many protein kinases to be differentially expressed. Among these, plant Ca2^+^ sensors. These included calmodulin (CaM), calcineurin B-like proteins (CBLs), CBL-interacting protein kinases (CIPKs), and Ca2^+^-dependent protein kinases (CDPKs). CDPKs are Ca^2+^ sensors unique to higher plants. They are structurally different from Ca^2+^-regulated protein kinases in other species [75]. The CDPKs have been proven to be involved in regulating plant responses to environmental stresses in different species [76], [77]. CIPK23 has been shown to directly phosphorylate the K^+^ transporter AtAKT1. CIPK23 is activated by the binding of two calcineurin B-like proteins, CBL1 and CBL9 [78]. The CBL1/9–CIPK23 pathway efficiently improves the activity of AKT1 and enhances K^+^ uptake under low-K stress conditions. The tag representing the CBL1/9 gene (Glyma05g05580-2.10-fold), CIPK23 gene (Glyma14g04430-1.83-fold), and CDPKs (Glyma14g02680-16.23-fold, Glyma04g38150-2.31-fold, and Glyma14g00320-2.47-fold) was found to be up-regulated in both varieties. We hypothesize that these genes might make an important contribution to adaptation to low-K stress conditions in soybeans. In addition, wall-associated kinases are also involved in various processes in plants, including pathogen resistance, heavy-metal tolerance, and organ development [79]. However, their role in tolerance to potassium deficiency is only minimally understood. In our DGE results, three genes encoding the wall-associated kinases were found to be up-regulated under low-K stress conditions. Meanwhile, two genes encoding MAP-kinase (Glyma17g06020, Glyma15g18860) were up-regulated in the low-K-tolerant variety. Recent investigations have confirmed major roles for defined MAPK pathways in plant development, cell proliferation, and hormone physiology, and both biotic and abiotic stress signaling [80]. These genes might play an important role in plant growth and development as well as in hormone and stress responses, and might be important for adaptation to low-K stress condition in soybeans.

Several other regulated genes were observed under low-K stress conditions. The ethylene response protein (Glyma16g01500–2.24-fold) showed short-term up-regulation of expression levels in the roots. Ethylene regulates many cellular and developmental processes in plants through a signaling pathway conserved in monocots and dicots [81]. Reactive oxygen species (ROS) are known to be involved in low-potassium signaling pathways [52]. Some reports have shown that ethylene acts upstream of reactive oxygen species in response to potassium deprivation [82]. Low-K^+^ inducible HAK5 expression is dependent on the production of ROS [52]. HAK5 also has an important role in the inducible high-affinity K^+^ uptake system in *Arabidopsis* roots [83]. These observations suggest the possibility that the ethylene-ethylene response protein-ROS-HAK5-dependent pathways are involved in low-K^+^-induced plant responses and that the ethylene response protein is a key element in the integration of signals for the regulation of defense response genes.

Five genes were predicted to be members of the ATP binding cassette (ABC) transporter family. All five were differentially expressed between the two varieties. The ABC superfamily is a large, diverse group of ubiquitous proteins, most of which mediate transport across biological membranes. ABC transporters have been shown to function not only as ATP-dependent pumps, but also as ion channels and channel regulators [84]. Several plant ABC genes have been found to participate in the abiotic stress response [85]. These genes might be helpful in investigating the mechanism of the response to low-K stress conditions.

One gene encoding BURP domain protein (Glyma14g20440) was found to be differentially expressed in the two varieties. We identified all 23 members of the BURP family in soybeans and found 17 to be stress-responsive, responding to at least one of the three stress treatments (ABA, NaCI, PEG) [86]. Previous research revealed that BURP-domain protein family genes might be important for plants responding to stress conditions. The soybean BURP gene may be K-responsive to low-K stress condition. Plants have evolved diverse adaptive physiological and biochemical mechanisms to resist various stressors. Consequently, that stress can alter the expression levels of related genes.

The present study investigated a large number of transcripts and identified a large number of differentially expressed genes related to the mechanisms of potassium regulation in two soybean genotypes under low-K stress conditions. We used Solexa/Illumina deep-sequencing technology. However, many ambiguous tags and unannotated genes were discounted. Future improvements to the knowledge base of soybean genome sequences may resolve some of these ambiguities, leading to the discovery of large numbers of genes related to potassium regulation. Further verification of these differentially expressed genes via transgenic technology could lead to improvement of the soybean’s resistance to low-K stress condition and its KUE.

## Materials and Methods

### Selection of Plant Materials and Growth Conditions

The identification of a low-K tolerant variety was an important step in our study. Forty soybean varieties (lines) were collected from the Oil Crops Research Institute at the Chinese Academy of Agricultural Science (Table S1). Sterilized seeds were pre-germinated on moistened filter paper in a plant growth chamber at 60% humidity and 28°C, under a 16/8 h (light/dark) cycle for 3–4 days. Then the seedlings were transferred into plastic pots with 1/2 strength modified Hoagland nutrient solution (2 mM Ca(NO_3_)_2_·4H_2_O, 2.5 mM KNO_3_, 0.5 mM NH_4_NO_3_, 0.5 mM KH_2_PO_4_, 1 mM MgSO_4_·7H_2_O, 0.05 mM Fe-EDTA, 0.005 mM KI, 0.1 mM H_3_BO_3_, 0.1 mM MnSO_4_·H_2_O, 0.03 mM ZnSO_4_·7H_2_O, 0.0001 mM CuSO_4_·5H_2_O, 0.001 mM Na_2_MO_4_·2H_2_O, 0.0001 mM CoCl_2_·6H_2_O). Their cotyledons were removed to eliminate any additional supply of nutrition. When the plants reached the 2-leaf stage, they were treated with 1/2 Hoagland nutrient solution with different concentrations of K^+^ (K1 level: 0.2 mmol L^−1^, K2 level: 0.5 mmol L^−1^, K3 level: 1.0 mmol L^−1^, K4 level: normal K concentration). The low K nutrient solution was made by a modification of 1/2 Hoagland nutrient solution: the KNO_3_ and KH_2_PO_4_ in the 1/2 Hoagland nutrient solution were replaced by NH_4_NO_3_ and NH_4_H_2_PO_4_, respectively; the final [K^+^] was adjusted by adding KCI [87]. The culture solution was refreshed every 4 days. We sampled the plant tissue at 6-day intervals. Stress symptoms (yellow leaves and small plants) were observed within 12 days. Different parts of the plant were sampled and dried to assess plant dry weight and potassium content using an inductively coupled plasma emission spectrometer for preliminary evaluation of low-K-tolerant soybean varieties. This experiment was conducted once. The relevant formulas are as follows [88]: (1) K accumulation  =  K content × dry weight; (2) K use efficiency (KUE): dry weight (g) ×1000/K content (mg/g).

In our experimental treatment, four K^+^ concentrations (K1, K2, K3 and K4) were selected for our nutrient solution. Among these concentrations, K4 represented a level sufficient for normal plant growth [89]. K1, K2 and K3 here represent the low-K^+^ level for plant growth. We found that an excessively low K^+^ concentration (K1 level) could lead to abnormal growth and plant death. We used K2 and K4 to represent low-K (-K) and high-K (+K) conditions.

After preliminary screening experiments, three low-K-tolerant varieties and two low-K-sensitive varieties were observed. We selected seeds from You06-71 (low-K-tolerant) and HengChun04-11 (low-K-sensitive) soybean varieties and grew then in 1/2 Hoagland solution. When plants reached the 2-leaf stage, they were treated with 1/2 Hoagland nutrient solution at K2 and K4 levels. The roots and shoots were harvested separately after 0.5, 2, 6, and 12 h and after 3, 6, 9, and 12 days of this treatment. Each sample was derived from at least three independent plants and three biological repeats. The plant tissues were frozen in liquid nitrogen and kept at –80°C until RNA isolation. Total RNA was extracted from the root and shoot samples using the method described by Liu in 2006 [90].

### Library Construction and Sequencing

At least 10 µg of total RNA (≥300 ng/µl) was sent to the Beijing Genomics Institute for Solexa sequencing (commercial service). The experimental process included sample preparation and sequencing. Ten micrograms of total RNA was extracted, and oligo (dT) magnetic adsorption beads were used to purify mRNA. These were then used as primers to synthesize the first and second-strand cDNA. The 5′ ends of the tags were generated using two types of endonuclease: NIaIII and DpnII. The bead-bound cDNA was then digested with restriction enzyme NlaIII, which recognizes and cuts off the CATG sites. All non-3′ cDNA fragments connected to oligo beads were washed away and an Illumina adaptor1 was ligated to the sticky 5′ end of each digested bead-bound cDNA fragment. The junction of the Illumina adaptor1 and the CATG site is the recognition site of MmeI, which is a type of endonuclease with separate recognition and digestion sites. It cuts 17 bp downstream of the CATG site, producing tags with adaptor1. After the removal of the 3′ fragments through magnetic bead precipitation, an Illumina adaptor2 was ligated to the 3′ ends of the tags, producing tags with different adaptors at both ends to form a tag library. After 15 cycles of linear PCR amplification, 95 bp fragments were purified by 6% TBE PAGE gel electrophoresis. After denaturation, the single-chain molecules were fixed onto the Illumina Sequencing Chip (flow cell). Each molecule grew into a single-molecule cluster-sequencing template through in situ amplification. Then four types of nucleotides, each labeled with four colors, were added and sequencing was performed by synthesis (SBS). Each tunnel generated millions of raw reads with a length of 35 bp. Then the raw data (tag sequences and counts) were produced. The raw sequences had 3′ adaptor fragments as well as a few low-quality sequences and several types of impurities. Raw sequences were transformed into clean tags after certain data-processing steps: (1) Removal of 3′ adaptor sequence: Because tags are only 21 bp long and the sequencing reads are 35 bp long, raw sequences include 3′ adaptor sequences. (2) Removal of empty reads (reads with only 3′ adaptor sequences but no tags). (3) Low-quality tags removal (tags with unknown sequences “N”). (4) Removal of tags that are too long or too short, leaving tags of exactly 21 bp. (5) Removal of tags with a copy number of 1 (likely sequencing error). (6) Generation of clean tags. “Clean tags” here refers to the tags used for analysis after filtering the dirty tags. “Distinct tags” include all types of clean tags. The underlying principles and specific step of tag preparation are shown in Table S14.

### Data Processing and Digital Tag Profiling

Sequencing-received raw image data was transformed by base calling into the sequence data, which is called raw data or raw reads, and it is stored in FASTQ format. These types of files store information about read sequences and quality. Each read is described in four lines in FASTQ files. Raw sequence reads were filtered by the Illumina pipeline. Raw sequences have 3′ adaptor fragments, a few low-quality sequences, and several types of impurities. All low-quality tags, such as short tags (<21 nt), empty reads, overly long tags, and tags with only a single copy were removed. Raw sequences were transformed into clean tags after data processing. To map DGE tags to the soybean genome (*Glycine max*), we created virtual libraries containing all the possible sequences 17 bases in length that were located next to a NIaIII restriction site (CATG). These high-quality sequences were mapped to genome reference sequences (http://phytozome.net/) (Phytozome V6.0). To monitor mapping events on both strands, the sense and complementary anti-sense sequences were included in the data collection process. During mapping, any transcript detected more than once during these experiments was excluded.

### Gene Annotation

Currently, the full genome sequence for the soybean (*Glycine max*) is available (http://www.soybase.org/). We used it to align and identify clean sequencing reads. Virtual libraries were built containing all possible CATG+17-base sequences in reference gene sequences. All clean tags were mapped to the reference sequences and only mismatches of no more than 1 bp were considered. Clean tags mapped to reference sequences from multiple genes were filtered. The remaining clean tags were designed to be unambiguous. The number of unambiguous clean tags per gene was calculated and then normalized to TPM. In this way, we have provided a more accurate and scientifically sound means of measuring the level of gene expression in these two varieties under low-K stress conditions.

### Statistical Evaluation of Differential Gene Expression

Statistical comparison was performed to identify differentially expressed genes between the libraries using the rigorous algorithm method described by Audic [24]. Gene expression was normalized to transcripts per million (TPM) clean tags. For gene expression variance, T-testing was performed to determine significant differences. *P* values corresponding to differential gene expression were assessed. The false discovery rate (FDR) method was used to determine the threshold *P* value through a multiple test and analysis as per Benjamini [91]. We used FDR≤0.001 and the absolute value of |log2Ratio|≥1 as a threshold to judge the significance of differences in gene expression. More stringent criteria, using smaller FDR values and bigger fold-change values, were used to identify DEGs.

### Quantitative RT-PCR Analysis

Analysis of the gene expression was performed via real-time PCR as described previously [92]. Evidence shows that transcription levels of housekeeping genes can vary considerably in response to changes in experimental conditions and across different types of tissue types [93], [94]. For this reason, we selected five housekeeping genes to evaluate the levels of gene expression. These five genes were TUA5, ACT2/7, UBQ10, UKN2, and HDC. The stability of housekeeping gene expression was analyzed with geNorm software (V3.50). We determined the value of stability measure (M) via a stepwise exclusion of the least stable housekeeping gene, created a stability ranking, and then used the relatively stable housekeeping gene to normalize the level of selected gene expression [95]. Selected gene sequences were obtained from the soybean sequence database. Primers were designed using Primer Premier 5.0 software (Premier Biosoft International, Palo Alto, CA, U.S.) and were synthesized commercially (Shanghai Sangon Biological Engineering Technology & Services Co., Ltd., Shanghai, China). RT-PCR was conducted with gene-specific primers and housekeeping gene primers (Table S11). real-time PCR reactions were performed with a Bio-Rad real-time thermal cycling system (Bio-Rad, Hercules, CA, U.S.) using SYBR-Green to assess gene expression. The reaction mixture (20 µl) contained 0.5 µl of each primer and the appropriate amounts of enzymes, cDNA, and fluorescent dyes. All runs used a negative control without any additional target cDNA, resulting in a lack of detectable fluorescence. A range of five dilutions of the total cDNA was tested under the same conditions. Amplification reactions were initiated with a pre-denaturing step at 95°C for 10 s followed by 40 cycles of denaturing (95°C for 5 s), annealing (55°C for 10 s), and extension (72°C for 15 s) during the second stage, and a final stage of 55°C to 95°C to determine the dissociation curves of the amplified products. All reactions were performed with at least three replicates. The relative quantitation of gene expression was calculated and normalized to housekeeping genes. Data were analyzed using Bio-Rad CFX Manager software and MS Excel 2003.

## Supporting Information

Figure S1 **Sequencing saturation analysis of the eight libraries in (A) HengChun04-11 and (B) You06-71.** The number of detected genes was found to increase as the total number of tags increased.
(DOC)

Table S1 **Soybean varieties used for screening.**
(XLS)

Table S2 **Expression of anti-sense genes detected from the eight libraries.**
(XLS)

Table S3 **Parts of pathways used for the DEGs.**
(DOC)

Table S4 **Unknown genes differentially expressed in the roots and shoots of two varieties of soybeans under low-K stress conditions.**
(XLS)

Table S5 **Differentially expressed genes involved in the soybean roots (** ***Glycine max*** **) and shoot development under low K stress conditions.**
(XLS)

Table S6 **Predicted genes differentially expressed in the roots and shoots of two varieties of soybeans under low-K stress conditions.**
(XLS)

Table S7 **Differentially expressed genes found in both roots and shoots.**
(XLS)

Table S8 **Transcription factors involved in genes differentially expressed in the roots and shoots of two varieties of soybeans under low-K stress conditions.**
(XLS)

Table S9 **Protein kinases involved in genes differentially expressed in the roots and shoots of two varieties of soybeans under low-K stress conditions.**
(XLS)

Table S10 **Oxidative stress-related genes in differentially expressed in the roots and shoots of two varieties of soybeans under low K stress conditions.**
(XLS)

Table S11 **Real-time PCR-primers for validated gene expression in results of DGEs and housekeeping genes.**
(XLS)

Table S12 **Evaluation of stable of housekeeping genes with geNorm software.**
(XLS)

Table S13 **Standardized calculation of the selected gene.**
(XLS)

Table S14 **Principle and step of tag preparation.**
(DOC)
