# Supplementary material for: Transcript Profile of the Response of Two Soybean Genotypes to Potassium Deficiency
Source: PLoS One. 2012 Jul 5;7(7):e39856. doi: 10.1371/journal.pone.0039856 (PMC3390323; doi:10.1371/journal.pone.0039856)
Supplement: Figure S1 — Sequencing saturation analysis of the eight libraries in (A) HengChun04-11 and (B) You06-71. The number of detected genes was found to increase as the total number of tags increased. (DOC) [file pone.0039856.s001.doc]

**A**

1

2

3

4

B

1

2

3

4

**Supplemental Figure S1.** Sequencing saturation analysis of the eight libraries in (A) HengChun04-11 and (B) You06-71. The number of detected genes was found to increase as the total number of tags increased.

(A1; L2, A2; L4, A3; L6, A4; L8)

(B1; L1, B2; L3, B3; L5, B4; L7)
