# Supplementary material for: Transcript Profile of the Response of Two Soybean Genotypes to Potassium Deficiency
Source: PLoS One. 2012 Jul 5;7(7):e39856. doi: 10.1371/journal.pone.0039856 (PMC3390323; doi:10.1371/journal.pone.0039856)
Supplement: Table S3 — Parts of pathways used for the DEGs. (DOC) [file pone.0039856.s004.doc]

**Supplemental table S3.** Parts of pathways used for the DEGs.

**Metabolic pathways (1435 members)**

Glyma03g39930, Glyma13g23060, Glyma02g47460, Glyma16g19350, Glyma08g20490, Glyma13g30560, Glyma08g07960, Glyma10g42870, Glyma01g36080, Glyma17g34570, Glyma13g07130, Glyma18g14410, Glyma06g38160, Glyma15g39090, Glyma05g34830, Glyma07g31280, Glyma07g17180, Glyma07g09430, Glyma11g04990, Glyma18g45900, Glyma12g09100, Glyma01g27900, Glyma02g14860, Glyma18g47710, Glyma03g37550, Glyma03g02120, Glyma08g14880, Glyma19g32880, Glyma08g11670, Glyma10g04790, Glyma10g43760, Glyma06g48340, Glyma13g06710, Glyma19g42830, Glyma16g22630, Glyma08g15840, Glyma13g37510, Glyma13g33300, Glyma10g00570, Glyma06g13880, Glyma10g04730, Glyma07g38620, Glyma03g38740, Glyma03g01630, Glyma15g06730, Glyma16g33880, Glyma09g34990, Glyma15g40140, Glyma08g09860, Glyma18g44320, Glyma09g34410, Glyma19g34900, Glyma05g29830, Glyma01g09660, Glyma05g08870, Glyma10g26970, Glyma08g44140, Glyma12g02200, Glyma05g31650, Glyma16g02400, Glyma11g15810, Glyma10g05030, Glyma16g24420, Glyma11g37580, Glyma03g40050, Glyma08g22920, Glyma19g30350, Glyma08g09300, Glyma13g23010, Glyma06g48030, Glyma10g27800, Glyma08g39370, Glyma04g05110, Glyma07g39620, Glyma19g29330, Glyma14g06460, Glyma04g13600, Glyma06g11860, Glyma03g40870, Glyma17g14630, Glyma04g39000, Glyma14g15170, Glyma18g03020, Glyma13g33730, Glyma08g14670, Glyma15g03130, Glyma02g34740, Glyma08g18750, Glyma04g01350, Glyma14g36420, Glyma11g07980, Glyma08g14730, Glyma01g38180, Glyma09g08920, Glyma02g47980, Glyma15g11810, Glyma09g31890, Glyma07g39870, Glyma15g00450, Glyma10g05090, Glyma13g44140, Glyma08g03190, Glyma14g37260, Glyma1246s00210, Glyma13g27490, Glyma17g36790, Glyma13g34680, Glyma08g07500, Glyma06g23560, Glyma08g33510, Glyma11g19430, Glyma13g16590, Glyma06g03220, Glyma10g12560, Glyma20g28620, Glyma18g10570, Glyma01g23180, Glyma05g00510, Glyma17g37540, Glyma17g08150, Glyma05g35930, Glyma06g03040, Glyma04g00960, Glyma08g09040, Glyma13g36090, Glyma18g04040, Glyma08g14900, Glyma0428s00200, Glyma10g07480, Glyma10g39540, Glyma03g37130, Glyma08g19170, Glyma14g30280, Glyma15g41640, Glyma17g31730, Glyma10g42800, Glyma15g01120, Glyma10g39300, Glyma07g13760, Glyma07g33020, Glyma07g03920, Glyma02g04770, Glyma11g06380, Glyma03g37280, Glyma08g41050, Glyma10g12530, Glyma03g21600, Glyma08g02630, Glyma07g14340, Glyma06g25360, Glyma13g31530, Glyma05g34500, Glyma05g19580, Glyma08g28530, Glyma09g36650, Glyma13g31590, Glyma19g40100, Glyma04g34480, Glyma17g21530, Glyma12g09780, Glyma05g38490, Glyma17g13740, Glyma17g09860, Glyma10g09880, Glyma08g39980, Glyma08g09180, Glyma15g13710, Glyma18g53430, Glyma16g01990, Glyma01g38110, Glyma18g15520, Glyma17g11720, Glyma06g06480, Glyma10g36990, Glyma13g32910, Glyma08g12930, Glyma20g12230, Glyma18g19360, Glyma16g27270, Glyma08g00470, Glyma17g37890, Glyma09g21900, Glyma15g10440, Glyma03g42250, Glyma09g27490, Glyma1000s00210, Glyma13g43370, Glyma15g09330, Glyma15g18410, Glyma20g20300, Glyma07g17720, Glyma07g14950, Glyma02g26390, Glyma15g39150, Glyma19g05580, Glyma11g24620, Glyma20g26110, Glyma15g08680, Glyma12g09810, Glyma02g47560, Glyma15g41690, Glyma07g31270, Glyma11g00580, Glyma17g03760, Glyma09g28200, Glyma20g32030, Glyma03g24980, Glyma02g14140, Glyma19g31440, Glyma08g03700, Glyma16g25860, Glyma10g07220, Glyma11g36140, Glyma13g42530, Glyma13g25570, Glyma08g20220, Glyma07g30440, Glyma11g18330, Glyma13g42340, Glyma09g41440, Glyma12g33790, Glyma03g27740, Glyma01g37630, Glyma11g07250, Glyma08g05190, Glyma16g27990, Glyma08g06820, Glyma17g00700, Glyma04g02300, Glyma09g36740, Glyma17g36090, Glyma02g01190, Glyma14g02630, Glyma07g01250, Glyma01g01370, Glyma13g06880, Glyma08g18800, Glyma01g38870, Glyma02g16390, Glyma03g42140, Glyma05g27430, Glyma09g11460, Glyma18g52810, Glyma11g10750, Glyma09g02590, Glyma17g14750, Glyma16g06250, Glyma15g41540, Glyma18g50870, Glyma10g25710, Glyma07g31660, Glyma17g14320, Glyma01g41190, Glyma03g40720, Glyma01g10080, Glyma19g28240, Glyma15g15310, Glyma01g38750, Glyma13g36700, Glyma07g02240, Glyma03g14960, Glyma09g07100, Glyma04g43360, Glyma11g33110, Glyma19g34120, Glyma14g39050, Glyma13g08010, Glyma12g32970, Glyma05g28610, Glyma18g42310, Glyma02g15630, Glyma18g53450, Glyma13g31580, Glyma08g46630, Glyma08g03060, Glyma05g26080, Glyma06g15940, Glyma14g08080, Glyma02g05350, Glyma13g03650, Glyma07g18280, Glyma09g27140, Glyma18g48820, Glyma18g02670, Glyma01g41310, Glyma14g23710, Glyma10g00820, Glyma13g29410, Glyma02g16000, Glyma01g43630, Glyma07g05820, Glyma13g07800, Glyma19g28900, Glyma02g13260, Glyma02g07940, Glyma07g19530, Glyma03g35170, Glyma13g05440, Glyma08g07880, Glyma10g39460, Glyma01g41430, Glyma20g07060, Glyma10g38890, Glyma06g45950, Glyma06g01850, Glyma02g13840, Glyma13g18080, Glyma15g41740, Glyma04g08880, Glyma13g33910, Glyma13g03280, Glyma14g00590, Glyma02g45190, Glyma10g43990, Glyma16g34360, Glyma09g38610, Glyma20g38200, Glyma14g02430, Glyma11g15900, Glyma17g07480, Glyma08g10870, Glyma18g43130, Glyma05g26140, Glyma15g40940, Glyma02g42470, Glyma20g10240, Glyma14g01730, Glyma05g27970, Glyma04g02510, Glyma11g36080, Glyma06g05520, Glyma19g05570, Glyma05g32190, Glyma17g14730, Glyma13g33290, Glyma16g17720, Glyma17g15500, Glyma10g44170, Glyma04g42000, Glyma20g39250, Glyma08g07140, Glyma13g27650, Glyma12g00730, Glyma19g44010, Glyma19g36070, Glyma07g34250, Glyma14g02860, Glyma02g14000, Glyma09g02840, Glyma17g34300, Glyma13g24120, Glyma06g11480, Glyma03g02390, Glyma13g40200, Glyma04g02230, Glyma02g00780, Glyma09g16810, Glyma13g33890, Glyma05g04940, Glyma15g12100, Glyma11g36690, Glyma10g03750, Glyma19g34410, Glyma12g32370, Glyma13g18800, Glyma15g03050, Glyma12g02270, Glyma05g00620, Glyma13g32830, Glyma06g45910, Glyma14g03410, Glyma12g23150, Glyma08g01870, Glyma17g06090, Glyma03g07460, Glyma11g02030, Glyma18g01280, Glyma05g37170, Glyma08g03240, Glyma17g10590, Glyma13g19880, Glyma15g42080, Glyma06g02550, Glyma03g35020, Glyma02g34760, Glyma03g41120, Glyma06g48360, Glyma16g26940, Glyma06g07110, Glyma07g38740, Glyma16g04530, Glyma04g12510, Glyma12g36380, Glyma03g29950, Glyma12g32380, Glyma13g18930, Glyma09g34460, Glyma19g37210, Glyma11g01840, Glyma09g25470, Glyma02g06830, Glyma20g29270, Glyma11g19400, Glyma15g00390, Glyma02g14450, Glyma09g40640, Glyma08g17120, Glyma07g03910, Glyma06g01210, Glyma09g38110, Glyma07g10170, Glyma14g21220, Glyma01g29470, Glyma12g32170, Glyma10g30880, Glyma08g46620, Glyma08g21370, Glyma09g20270, Glyma07g16310, Glyma15g14140, Glyma11g37280, Glyma03g38350, Glyma11g14390, Glyma12g32000, Glyma15g13860, Glyma02g46970, Glyma20g35340, Glyma02g10150, Glyma03g04760, Glyma01g34360, Glyma05g08990, Glyma20g29230, Glyma15g22780, Glyma05g37840, Glyma05g00910, Glyma13g31850, Glyma02g25300, Glyma11g25900, Glyma06g12350, Glyma19g44790, Glyma08g36580, Glyma04g05190, Glyma07g00900, Glyma11g38130, Glyma06g15030, Glyma15g07720, Glyma05g22300, Glyma10g33520, Glyma07g09640, Glyma12g01690, Glyma01g33480, Glyma07g00890, Glyma05g26660, Glyma07g05750, Glyma01g00980, Glyma09g02610, Glyma07g00870, Glyma20g24810, Glyma20g31190, Glyma08g13170, Glyma08g16980, Glyma12g35050, Glyma09g32430, Glyma05g27890, Glyma03g11610, Glyma13g15560, Glyma11g14160, Glyma11g09330, Glyma04g34960, Glyma05g17410, Glyma06g40790, Glyma13g16950, Glyma05g30270, Glyma19g37830, Glyma08g16770, Glyma01g01800, Glyma13g07110, Glyma18g04940, Glyma18g42300, Glyma07g15150, Glyma03g17950, Glyma03g29560, Glyma17g00910, Glyma18g04950, Glyma20g38590, Glyma20g37660, Glyma13g19070, Glyma09g39850, Glyma10g29380, Glyma08g01480, Glyma13g31570, Glyma18g44060, Glyma12g09930, Glyma20g32770, Glyma09g32170, Glyma16g34570, Glyma01g36440, Glyma20g25830, Glyma09g02900, Glyma05g23220, Glyma12g03740, Glyma09g18450, Glyma02g39630, Glyma18g18210, Glyma13g07630, Glyma15g39750, Glyma12g04220, Glyma01g03820, Glyma08g02100, Glyma12g29590, Glyma12g04880, Glyma13g43980, Glyma19g31330, Glyma14g36280, Glyma02g04310, Glyma12g07390, Glyma04g39190, Glyma18g48370, Glyma17g15940, Glyma08g09660, Glyma15g07340, Glyma01g00730, Glyma16g04630, Glyma13g30490, Glyma16g25390, Glyma07g29670, Glyma07g01220, Glyma05g31450, Glyma06g11460, Glyma04g16260, Glyma02g38160, Glyma08g18900, Glyma07g06950, Glyma19g02690, Glyma11g12870, Glyma05g30600, Glyma18g02210, Glyma08g11620, Glyma17g15320, Glyma18g12920, Glyma08g28430, Glyma01g36700, Glyma02g04780, Glyma01g02330, Glyma16g33560, Glyma17g01300, Glyma03g36910, Glyma15g03040, Glyma11g09930, Glyma08g09670, Glyma09g02030, Glyma05g07020, Glyma12g00780, Glyma03g31580, Glyma12g32870, Glyma03g37410, Glyma19g32660, Glyma13g21120, Glyma15g00400, Glyma10g38350, Glyma06g01600, Glyma08g45990, Glyma05g26240, Glyma06g02310, Glyma19g04250, Glyma08g09170, Glyma10g38590, Glyma05g32130, Glyma06g16750, Glyma02g08050, Glyma07g08970, Glyma11g35290, Glyma18g06190, Glyma19g03670, Glyma16g33030, Glyma06g36210, Glyma15g10890, Glyma08g22480, Glyma05g31390, Glyma03g39510, Glyma13g01170, Glyma06g15140, Glyma06g11120, Glyma20g17440, Glyma20g31250, Glyma18g01500, Glyma18g51420, Glyma05g38570, Glyma13g42330, Glyma09g01630, Glyma07g36610, Glyma13g29390, Glyma01g07780, Glyma06g11920, Glyma10g36380, Glyma13g37630, Glyma15g15020, Glyma07g33090, Glyma18g06250, Glyma10g05530, Glyma16g18380, Glyma07g00910, Glyma05g01300, Glyma06g15540, Glyma19g32850, Glyma14g15360, Glyma13g35480, Glyma09g07430, Glyma08g18510, Glyma12g01780, Glyma18g53100, Glyma04g43070, Glyma15g07710, Glyma13g00910, Glyma09g02650, Glyma07g07580, Glyma17g36940, Glyma03g34670, Glyma05g33310, Glyma13g01360, Glyma04g03740, Glyma10g20570, Glyma07g38260, Glyma17g16840, Glyma08g01750, Glyma13g29130, Glyma16g04940, Glyma01g42420, Glyma08g03730, Glyma11g35300, Glyma05g27290, Glyma04g34670, Glyma11g12410, Glyma06g05280, Glyma02g37020, Glyma11g10770, Glyma05g15230, Glyma07g36150, Glyma15g00780, Glyma07g02640, Glyma08g20690, Glyma07g03930, Glyma16g33820, Glyma13g24110, Glyma02g13850, Glyma16g01650, Glyma04g10100, Glyma06g19750, Glyma14g04610, Glyma20g02850, Glyma08g01770, Glyma20g24200, Glyma15g02850, Glyma10g39470, Glyma12g33350, Glyma14g37040, Glyma09g02670, Glyma09g36280, Glyma08g06300, Glyma20g31110, Glyma16g32220, Glyma17g03990, Glyma01g26750, Glyma14g08400, Glyma15g00600, Glyma17g06280, Glyma14g37680, Glyma08g47880, Glyma16g27060, Glyma19g22690, Glyma11g07670, Glyma11g11960, Glyma06g04190, Glyma17g04920, Glyma07g39020, Glyma04g00950, Glyma11g08230, Glyma09g04060, Glyma01g16610, Glyma09g11820, Glyma01g37540, Glyma05g36350, Glyma06g12770, Glyma05g10140, Glyma09g08270, Glyma01g03590, Glyma10g32260, Glyma07g09370, Glyma18g13260, Glyma04g03590, Glyma02g03870, Glyma10g03440, Glyma13g36110, Glyma07g11610, Glyma02g45780, Glyma08g20080, Glyma02g10360, Glyma11g20730, Glyma11g31800, Glyma17g38220, Glyma03g37670, Glyma16g27220, Glyma02g07250, Glyma19g05980, Glyma12g02450, Glyma01g04470, Glyma08g02050, Glyma11g03810, Glyma13g17580, Glyma02g40040, Glyma03g28690, Glyma02g42730, Glyma09g38010, Glyma12g31920, Glyma16g28310, Glyma13g38300, Glyma02g41850, Glyma15g19670, Glyma16g09020, Glyma20g28300, Glyma16g08990, Glyma12g31850, Glyma18g12090, Glyma13g31670, Glyma02g37350, Glyma14g20400, Glyma01g01060, Glyma13g17420, Glyma01g44900, Glyma15g11490, Glyma05g08270, Glyma13g38310, Glyma19g36990, Glyma01g43880, Glyma18g40210, Glyma19g00420, Glyma11g06510, Glyma07g21100, Glyma19g32950, Glyma20g11950, Glyma15g13560, Glyma19g28770, Glyma11g04210, Glyma16g32390, Glyma11g16010, Glyma20g38570, Glyma0103s00220, Glyma13g26190, Glyma02g40720, Glyma07g34030, Glyma05g26830, Glyma02g02020, Glyma18g38660, Glyma05g03340, Glyma13g28090, Glyma19g39680, Glyma18g47130, Glyma09g38260, Glyma09g41870, Glyma13g01500, Glyma12g02250, Glyma04g40920, Glyma19g14500, Glyma06g13280, Glyma14g11860, Glyma13g41520, Glyma10g34150, Glyma02g41820, Glyma09g38550, Glyma08g04460, Glyma11g30030, Glyma16g06070, Glyma11g37360, Glyma14g23910, Glyma09g18860, Glyma01g33150, Glyma01g41280, Glyma04g33050, Glyma17g06120, Glyma19g25300, Glyma06g37150, Glyma08g38510, Glyma14g04720, Glyma01g43660, Glyma06g02290, Glyma17g16020, Glyma14g11320, Glyma06g08850, Glyma14g01840, Glyma02g03270, Glyma20g25640, Glyma17g20620, Glyma13g07310, Glyma05g03140, Glyma09g07570, Glyma09g31250, Glyma05g00500, Glyma01g02570, Glyma19g32760, Glyma19g32650, Glyma09g27740, Glyma12g09800, Glyma12g01760, Glyma19g40000, Glyma10g06800, Glyma19g39270, Glyma07g00860, Glyma19g37340, Glyma05g04310, Glyma08g17470, Glyma15g13680, Glyma04g21910, Glyma05g06270, Glyma13g04710, Glyma20g14210, Glyma07g02180, Glyma17g24670, Glyma09g05170, Glyma17g35800, Glyma10g17850, Glyma17g04210, Glyma18g49110, Glyma18g47240, Glyma20g03780, Glyma19g40770, Glyma01g43780, Glyma09g30070, Glyma10g16060, Glyma07g38390, Glyma11g18990, Glyma05g01960, Glyma17g30800, Glyma18g49550, Glyma08g07110, Glyma02g05770, Glyma01g07860, Glyma17g14790, Glyma08g27600, Glyma13g31980, Glyma15g35410, Glyma20g23530, Glyma09g31840, Glyma04g08560, Glyma11g29920, Glyma19g01790, Glyma12g19960, Glyma07g35110, Glyma07g09000, Glyma05g25650, Glyma15g11310, Glyma04g24880, Glyma12g24200, Glyma16g24340, Glyma13g04690, Glyma11g01240, Glyma17g15070, Glyma19g30600, Glyma16g26130, Glyma12g04040, Glyma08g06110, Glyma18g16250, Glyma06g47670, Glyma15g25170, Glyma18g20800, Glyma09g28460, Glyma06g01460, Glyma18g01240, Glyma13g28910, Glyma18g32210, Glyma12g06480, Glyma07g17170, Glyma10g10160, Glyma04g01060, Glyma14g03200, Glyma16g21920, Glyma01g39460, Glyma11g01820, Glyma02g37080, Glyma13g06700, Glyma13g16560, Glyma15g07030, Glyma19g29180, Glyma20g37870, Glyma19g38860, Glyma02g31990, Glyma01g21710, Glyma20g18280, Glyma18g02690, Glyma06g13860, Glyma14g27660, Glyma12g12930, Glyma10g21100, Glyma03g08290, Glyma10g40110, Glyma18g12330, Glyma08g27630, Glyma13g42630, Glyma19g00900, Glyma07g03230, Glyma05g05460, Glyma20g06230, Glyma06g01950, Glyma11g08560, Glyma06g03160, Glyma09g02800, Glyma16g14490, Glyma02g27930, Glyma14g27640, Glyma07g34840, Glyma08g19070, Glyma04g03260, Glyma18g45650, Glyma08g37260, Glyma04g06990, Glyma02g46380, Glyma05g26040, Glyma11g35950, Glyma07g05690, Glyma18g48830, Glyma20g33090, Glyma10g42860, Glyma09g01170, Glyma19g41430, Glyma06g16770, Glyma19g42620, Glyma02g01170, Glyma09g29840, Glyma02g38990, Glyma11g14600, Glyma05g00590, Glyma02g14110, Glyma13g03750, Glyma01g20430, Glyma09g33320, Glyma18g10260, Glyma03g34760, Glyma01g41970, Glyma19g35780, Glyma03g38960, Glyma03g02550, Glyma09g03620, Glyma08g04880, Glyma07g36040, Glyma01g35680, Glyma13g42720, Glyma04g12150, Glyma01g34950, Glyma03g38030, Glyma19g42610, Glyma17g08820, Glyma10g39440, Glyma02g00440, Glyma0886s00200, Glyma11g35500, Glyma02g01140, Glyma13g21540, Glyma18g02520, Glyma11g33970, Glyma07g08980, Glyma06g17640, Glyma16g09250, Glyma10g28140, Glyma20g31890, Glyma13g31420, Glyma08g08130, Glyma02g45790, Glyma01g20460, Glyma12g31020, Glyma07g14030, Glyma19g01890, Glyma13g09400, Glyma04g11890, Glyma03g39360, Glyma18g08180, Glyma13g42420, Glyma12g17230, Glyma16g13830, Glyma06g08670, Glyma01g33650, Glyma01g40820, Glyma18g32710, Glyma07g06480, Glyma16g32960, Glyma04g39370, Glyma12g36140, Glyma16g06030, Glyma07g07280, Glyma12g15460, Glyma07g05420, Glyma11g17480, Glyma08g45100, Glyma13g34560, Glyma13g04590, Glyma14g05020, Glyma05g32910, Glyma04g21360, Glyma05g00530, Glyma08g02980, Glyma18g12740, Glyma05g03120, Glyma20g11110, Glyma10g02160, Glyma17g03500, Glyma14g38210, Glyma16g27480, Glyma08g20460, Glyma18g13610, Glyma05g27960, Glyma07g30510, Glyma08g36520, Glyma06g10130, Glyma10g34160, Glyma18g51440, Glyma17g02150, Glyma19g40020, Glyma01g33450, Glyma14g10550, Glyma10g02730, Glyma07g09090, Glyma02g01230, Glyma12g14230, Glyma11g01860, Glyma03g34120, Glyma11g21180, Glyma15g20180, Glyma15g03250, Glyma15g35290, Glyma19g03530, Glyma07g17880, Glyma14g37440, Glyma15g01300, Glyma01g36500, Glyma17g03080, Glyma11g07430, Glyma07g08040, Glyma05g37910, Glyma13g20170, Glyma05g03830, Glyma03g38000, Glyma16g04750, Glyma05g10880, Glyma01g41820, Glyma18g01330, Glyma07g39160, Glyma08g37270, Glyma16g26820, Glyma01g27710, Glyma18g16980, Glyma13g21230, Glyma08g06810, Glyma10g43850, Glyma06g46350, Glyma06g43050, Glyma03g01010, Glyma14g19220, Glyma19g08720, Glyma19g31280, Glyma03g27600, Glyma15g40890, Glyma20g28850, Glyma14g06760, Glyma17g04940, Glyma06g16110, Glyma18g48810, Glyma18g51640, Glyma08g18990, Glyma02g45940, Glyma06g45450, Glyma18g35220, Glyma01g39990, Glyma08g46160, Glyma09g40690, Glyma13g42140, Glyma14g39930, Glyma06g15350, Glyma09g35660, Glyma02g15470, Glyma02g14090, Glyma11g37820, Glyma09g33860, Glyma14g39070, Glyma09g36660, Glyma13g13040, Glyma12g00860, Glyma18g12390, Glyma07g03490, Glyma17g01580, Glyma03g21540, Glyma11g04650, Glyma14g01420, Glyma03g05070, Glyma06g03360, Glyma0048s00280, Glyma12g04980, Glyma02g11180, Glyma01g32360, Glyma03g41040, Glyma10g15520, Glyma08g14280, Glyma08g47380, Glyma18g43460, Glyma13g17570, Glyma16g22650, Glyma04g37360, Glyma06g21920, Glyma20g22430, Glyma18g01700, Glyma05g03990, Glyma08g09890, Glyma12g03060, Glyma16g03680, Glyma16g32650, Glyma20g39450, Glyma02g00810, Glyma05g28490, Glyma13g20390, Glyma15g03060, Glyma20g24830, Glyma19g25930, Glyma02g08390, Glyma07g32340, Glyma10g35380, Glyma04g09180, Glyma04g03780, Glyma03g28580, Glyma10g35660, Glyma07g33070, Glyma08g01820, Glyma07g32330, Glyma12g22280, Glyma10g40140, Glyma09g28100, Glyma05g36610, Glyma06g12780, Glyma08g08610, Glyma03g07380, Glyma12g10590, Glyma06g46180, Glyma06g33380, Glyma10g05800, Glyma05g32970, Glyma10g05610, Glyma08g10760, Glyma11g33160, Glyma02g39320, Glyma05g33790, Glyma11g08180, Glyma05g35350, Glyma06g16710, Glyma14g17670, Glyma03g23770, Glyma17g06080, Glyma05g36240, Glyma07g07810, Glyma20g33380, Glyma18g06230, Glyma01g00540, Glyma15g14330, Glyma14g34550, Glyma14g04110, Glyma04g00460, Glyma17g13930, Glyma11g02950, Glyma17g01610, Glyma02g13270, Glyma10g02480, Glyma10g32230, Glyma08g00540, Glyma13g44040, Glyma19g38390, Glyma09g31870, Glyma10g40870, Glyma16g32040, Glyma20g38170, Glyma02g02450, Glyma03g14450, Glyma13g36670, Glyma07g28910, Glyma11g35700, Glyma13g44970, Glyma09g34110, Glyma13g40960, Glyma09g36680, Glyma01g36680, Glyma07g32790, Glyma06g46190, Glyma13g40380, Glyma19g25980, Glyma10g30400, Glyma04g41990, Glyma13g23950, Glyma01g40980, Glyma05g30130, Glyma05g04300, Glyma07g05550, Glyma16g05400, Glyma08g04890, Glyma12g02030, Glyma01g11180, Glyma11g07240, Glyma19g06720, Glyma05g00220, Glyma17g38120, Glyma12g01920, Glyma13g25070, Glyma10g24590, Glyma11g00810, Glyma10g07590, Glyma17g13730, Glyma12g36150, Glyma18g52110, Glyma04g09510, Glyma11g08950, Glyma10g32660, Glyma12g06300, Glyma19g31220, Glyma08g03570, Glyma02g40470, Glyma09g32560, Glyma15g01170, Glyma17g10010, Glyma12g36100, Glyma08g03280, Glyma20g28720, Glyma15g41550, Glyma08g08630, Glyma06g23890, Glyma07g34610, Glyma06g03350, Glyma14g06070, Glyma01g41990, Glyma14g38290, Glyma20g38710, Glyma07g09110, Glyma02g28880, Glyma01g44280, Glyma18g39550, Glyma14g36850, Glyma08g19180, Glyma08g30150, Glyma15g12570, Glyma17g14330, Glyma11g06170, Glyma09g25070, Glyma06g18030, Glyma01g43540, Glyma07g13100, Glyma18g44350, Glyma11g36620, Glyma01g42920, Glyma11g19330, Glyma13g32720, Glyma09g01420, Glyma13g37570, Glyma05g37720, Glyma06g00230, Glyma03g01270, Glyma11g05140, Glyma12g30210, Glyma12g31770, Glyma14g05190, Glyma03g34740, Glyma12g35830, Glyma03g32140, Glyma15g01360, Glyma15g37200, Glyma01g07770, Glyma07g38570, Glyma04g37000, Glyma09g39870, Glyma13g11700, Glyma17g05980, Glyma03g38810, Glyma07g08050, Glyma06g17140, Glyma15g14440, Glyma04g03120, Glyma02g06710, Glyma12g04080, Glyma01g24520, Glyma11g29460, Glyma02g05440, Glyma08g26670, Glyma07g40340, Glyma01g37280, Glyma16g24610, Glyma04g10090, Glyma09g26770, Glyma13g34670, Glyma03g28510, Glyma10g34970, Glyma01g03260, Glyma10g30000, Glyma13g28590, Glyma03g03190, Glyma13g41960, Glyma15g12780, Glyma12g06320, Glyma11g33480, Glyma02g38730, Glyma13g33620, Glyma11g27480, Glyma06g42830, Glyma02g47340, Glyma01g43720, Glyma05g03470, Glyma20g28860, Glyma18g19660, Glyma07g16080, Glyma06g00510, Glyma12g12710, Glyma14g38180, Glyma03g40490, Glyma11g01230, Glyma08g22600, Glyma05g32070, Glyma02g47940, Glyma18g04730, Glyma15g37710, Glyma11g10130, Glyma05g33120, Glyma11g15020, Glyma13g05830, Glyma20g25790, Glyma05g04860, Glyma12g34920, Glyma18g38670, Glyma17g02260, Glyma17g14950, Glyma13g42150, Glyma18g07680, Glyma05g29740, Glyma08g17010, Glyma16g06850, Glyma13g24200, Glyma08g04370, Glyma17g05550, Glyma17g34960, Glyma13g39210, Glyma03g40880, Glyma02g47750, Glyma19g43150, Glyma17g12100, Glyma18g06840, Glyma15g07240, Glyma15g13910, Glyma05g22510, Glyma19g32410, Glyma10g25790, Glyma08g06840, Glyma19g36630, Glyma12g07740, Glyma05g32460, Glyma02g10570, Glyma14g24140, Glyma02g40290, Glyma05g25400, Glyma10g38900, Glyma17g07400, Glyma06g17990, Glyma17g38190, Glyma07g02260, Glyma19g01210, Glyma20g38950, Glyma18g02330, Glyma02g15640, Glyma09g00710, Glyma20g37610, Glyma02g40740, Glyma07g01280, Glyma08g15420, Glyma09g32390, Glyma11g35310, Glyma12g09490, Glyma14g02380, Glyma14g16190, Glyma17g14070, Glyma13g15140, Glyma01g38980, Glyma12g03650, Glyma03g40680, Glyma02g46540, Glyma05g35880, Glyma13g20800, Glyma04g30350, Glyma18g42280, Glyma20g29690, Glyma20g01060, Glyma05g08730, Glyma10g35750, Glyma01g01300, Glyma11g13590, Glyma12g10420, Glyma07g15360, Glyma05g04270, Glyma10g00920, Glyma01g33070, Glyma14g35370, Glyma11g06180, Glyma03g37250, Glyma01g36740, Glyma19g30880, Glyma16g03080, Glyma20g38780, Glyma05g33470, Glyma06g18110, Glyma04g38630, Glyma09g28120, Glyma10g33550, Glyma13g42270, Glyma17g37270, Glyma20g19000, Glyma17g29320, Glyma17g23500, Glyma06g34190, Glyma13g27740, Glyma18g03100, Glyma08g10840, Glyma15g04970, Glyma07g37570, Glyma17g11690, Glyma08g39330, Glyma02g45910, Glyma12g34910, Glyma11g11020, Glyma20g23670, Glyma10g08440, Glyma09g29610, Glyma18g52430, Glyma08g42430, Glyma03g02410, Glyma03g36210, Glyma10g41310, Glyma10g21840, Glyma02g27260

**[Flavonoid biosynthesis](../../../../D:%5C%E7%8E%8B%E7%A8%8B2012%E5%8D%9A%E5%A3%AB%E6%AF%95%E4%B8%9A%E5%AD%A3%5C%E7%8E%8B%E7%A8%8BPHD%5CD%5C%E7%8E%8B%E7%A8%8B%5C%E7%8E%8B%E7%A8%8B%E6%95%B0%E6%8D%AE%5C%E5%8D%8E%E5%A4%A7%E5%9F%BA%E5%9B%A0%E8%A1%A8%E8%BE%BE%E8%B0%B1%5C%E8%A1%A8%E8%BE%BE%E8%B0%B1%E7%BB%93%E6%9E%9C%5C%E5%8D%8E%E5%A4%A7%E5%9F%BA%E5%9B%A0%E7%BB%84%E6%9C%80%E7%BB%88%E7%BB%93%E6%9E%9C-%E6%A0%B9%5Ccomplete%5Cupload%5Cpathway%5Chengchungenxi0-5hvsyougenxi0-5h.htm#gene5%23gene5) (150 members)**

Glyma19g32880, Glyma13g06710, Glyma10g06990, Glyma13g33300, Glyma05g31650, Glyma16g02400, Glyma18g03020, Glyma13g33730, Glyma13g37830, Glyma20g28620, Glyma05g00510, Glyma08g09040, Glyma08g14900, Glyma09g24900, Glyma11g06380, Glyma04g22130, Glyma08g42500, Glyma16g01990, Glyma03g42250, Glyma17g37060, Glyma03g24980, Glyma10g07220, Glyma03g27740, Glyma01g38870, Glyma03g42140, Glyma18g50870, Glyma17g14320, Glyma05g28610, Glyma08g46630, Glyma05g26080, Glyma14g08080, Glyma07g18280, Glyma07g05820, Glyma02g43230, Glyma03g14210, Glyma02g13840, Glyma15g40940, Glyma02g42470, Glyma05g27970, Glyma07g34250, Glyma15g38670, Glyma13g33890, Glyma12g36380, Glyma03g29950, Glyma19g37210, Glyma02g14450, Glyma08g46620, Glyma08g01360, Glyma20g35340, Glyma19g44790, Glyma10g30120, Glyma11g07900, Glyma20g24810, Glyma02g39630, Glyma01g37810, Glyma06g41520, Glyma01g00730, Glyma08g11620, Glyma06g01420, Glyma14g07820, Glyma13g29390, Glyma08g42490, Glyma17g18840, Glyma17g36940, Glyma02g13850, Glyma16g32220, Glyma14g37680, Glyma06g04190, Glyma10g32260, Glyma13g36110, Glyma13g37810, Glyma11g31800, Glyma18g12280, Glyma11g03810, Glyma02g37350, Glyma01g43880, Glyma18g40210, Glyma20g38570, Glyma14g06280, Glyma05g26830, Glyma10g30110, Glyma12g02250, Glyma01g33150, Glyma17g16330, Glyma05g00500, Glyma19g32650, Glyma02g33100, Glyma13g04710, Glyma09g05170, Glyma13g37850, Glyma03g03340, Glyma13g37840, Glyma17g30800, Glyma12g34390, Glyma18g50330, Glyma09g31840, Glyma19g01790, Glyma16g24340, Glyma19g30600, Glyma16g29960, Glyma06g01460, Glyma04g01060, Glyma01g39460, Glyma19g43340, Glyma20g33090, Glyma18g10260, Glyma03g34760, Glyma19g03770, Glyma17g08820, Glyma07g05420, Glyma05g00530, Glyma18g13610, Glyma05g27960, Glyma08g36520, Glyma06g17590, Glyma10g43850, Glyma15g40890, Glyma0317s00200, Glyma18g35220, Glyma08g41930, Glyma06g21920, Glyma09g40590, Glyma09g40580, Glyma07g32340, Glyma04g03780, Glyma07g32330, Glyma16g04350, Glyma02g42180, Glyma03g23770, Glyma16g32040, Glyma07g28910, Glyma18g12180, Glyma06g04440, Glyma05g00220, Glyma07g09110, Glyma17g14330, Glyma07g13100, Glyma11g29460, Glyma09g26770, Glyma11g07510, Glyma13g24200, Glyma19g44360, Glyma04g04250, Glyma02g40290, Glyma06g03410, Glyma18g50340, Glyma02g18380, Glyma13g06230, Glyma17g11690, Glyma03g02410

**purine metabolism (118 members)**

Glyma08g44140, Glyma10g07480, Glyma15g41640, Glyma10g39300, Glyma07g33020, Glyma10g12530, Glyma08g02630, Glyma06g06480, Glyma16g27270, Glyma07g14950, Glyma11g36140, Glyma19g28900, Glyma03g35170, Glyma13g33910, Glyma04g42000, Glyma13g27650, Glyma14g02860, Glyma18g05580, Glyma03g07460, Glyma13g19880, Glyma16g04530, Glyma18g05590, Glyma01g29470, Glyma02g46970, Glyma20g29230, Glyma11g25900, Glyma01g00980, Glyma19g37830, Glyma03g29560, Glyma16g08390, Glyma12g04880, Glyma02g04310, Glyma12g07390, Glyma01g36700, Glyma05g08780, Glyma10g38350, Glyma10g38590, Glyma19g03670, Glyma20g17440, Glyma18g51420, Glyma10g05530, Glyma09g07430, Glyma11g10800, Glyma16g33820, Glyma09g36280, Glyma20g31110, Glyma09g11820, Glyma06g12770, Glyma01g03590, Glyma03g37670, Glyma01g44900, Glyma19g32950, Glyma13g26190, Glyma09g41870, Glyma13g41520, Glyma16g06070, Glyma05g03140, Glyma09g07570, Glyma05g04310, Glyma17g14790, Glyma12g19960, Glyma07g35110, Glyma15g11310, Glyma12g04040, Glyma14g03200, Glyma20g37870, Glyma12g12930, Glyma10g40110, Glyma08g37260, Glyma04g06990, Glyma05g26040, Glyma01g41970, Glyma13g31420, Glyma13g42420, Glyma04g21360, Glyma18g12740, Glyma05g03120, Glyma08g20460, Glyma16g04750, Glyma06g16110, Glyma18g51640, Glyma08g18990, Glyma06g15350, Glyma09g35660, Glyma02g15470, Glyma12g00860, Glyma0048s00280, Glyma19g25930, Glyma08g01820, Glyma10g05610, Glyma10g02480, Glyma10g32230, Glyma01g40980, Glyma08g04890, Glyma12g01920, Glyma09g32560, Glyma07g34610, Glyma18g39550, Glyma15g12570, Glyma19g29480, Glyma11g05140, Glyma03g34740, Glyma07g38570, Glyma13g34670, Glyma03g28510, Glyma02g17410, Glyma05g32070, Glyma01g34700, Glyma15g37710, Glyma19g32410, Glyma19g36630, Glyma08g45960, Glyma05g25400, Glyma11g37250, Glyma20g29690, Glyma10g33550, Glyma17g23500, Glyma02g45910

**ABC transporters (44 members)**

Glyma04g34130, Glyma14g38800, Glyma08g20780, Glyma03g29230, Glyma03g37200, Glyma10g37160, Glyma09g04980, Glyma19g35250, Glyma08g10710, Glyma13g43870, Glyma15g09900, Glyma07g36170, Glyma02g40490, Glyma05g32620, Glyma07g01860, Glyma09g27220, Glyma13g22250, Glyma16g21050, Glyma08g20770, Glyma18g09600, Glyma20g38610, Glyma19g39810, Glyma07g36160, Glyma03g24300, Glyma07g21050, Glyma05g31270, Glyma03g33250, Glyma10g27790, Glyma20g32580, Glyma12g02290, Glyma06g20370, Glyma11g09960, Glyma10g37150, Glyma20g30490, Glyma18g32860, Glyma16g08480, Glyma02g18670, Glyma14g15390, Glyma05g00240, Glyma10g08560, Glyma19g01980, Glyma17g04350, Glyma13g18960, Glyma07g12680

**[Ubiquitin mediated proteolysis](../../../../D:%5C%E7%8E%8B%E7%A8%8B2012%E5%8D%9A%E5%A3%AB%E6%AF%95%E4%B8%9A%E5%AD%A3%5C%E7%8E%8B%E7%A8%8BPHD%5CD%5C%E7%8E%8B%E7%A8%8B%5C%E7%8E%8B%E7%A8%8B%E6%95%B0%E6%8D%AE%5C%E5%8D%8E%E5%A4%A7%E5%9F%BA%E5%9B%A0%E8%A1%A8%E8%BE%BE%E8%B0%B1%5C%E8%A1%A8%E8%BE%BE%E8%B0%B1%E7%BB%93%E6%9E%9C%5C%E5%8D%8E%E5%A4%A7%E5%9F%BA%E5%9B%A0%E7%BB%84%E6%9C%80%E7%BB%88%E7%BB%93%E6%9E%9C-%E6%A0%B9%5Ccomplete%5Cupload%5Cpathway%5Chengchungenxi0-5hvsyougenxi0-5h.htm#gene17%23gene17) (140 members)**

Glyma06g37080, Glyma03g32070, Glyma10g33850, Glyma05g27240, Glyma09g25090, Glyma10g42850, Glyma02g45390, Glyma08g41050, Glyma11g02110, Glyma19g34640, Glyma16g22260, Glyma19g41840, Glyma18g15520, Glyma09g41500, Glyma14g03420, Glyma13g35190, Glyma19g34820, Glyma12g31500, Glyma16g06870, Glyma08g24480, Glyma15g10400, Glyma20g32340, Glyma13g11260, Glyma18g46990, Glyma02g42510, Glyma02g11480, Glyma12g25700, Glyma04g38670, Glyma04g02840, Glyma03g39290, Glyma11g06830, Glyma03g36270, Glyma06g14590, Glyma19g36400, Glyma13g22410, Glyma05g01980, Glyma14g37230, Glyma02g39500, Glyma19g02540, Glyma08g01940, Glyma08g15580, Glyma13g38890, Glyma17g09940, Glyma10g29000, Glyma02g41810, Glyma08g13680, Glyma15g09890, Glyma18g44850, Glyma07g37850, Glyma13g27180, Glyma18g26120, Glyma07g36390, Glyma13g19980, Glyma04g37620, Glyma08g27700, Glyma13g38720, Glyma10g10110, Glyma17g29800, Glyma06g15630, Glyma19g44520, Glyma07g31630, Glyma20g22300, Glyma07g16400, Glyma03g36490, Glyma13g09290, Glyma19g01340, Glyma18g06620, Glyma17g02630, Glyma13g28770, Glyma15g07050, Glyma02g02650, Glyma14g22680, Glyma06g13440, Glyma13g26890, Glyma08g00240, Glyma18g04130, Glyma05g27140, Glyma11g05780, Glyma11g05130, Glyma05g26360, Glyma15g08040, Glyma06g13270, Glyma02g11850, Glyma01g40170, Glyma13g04270, Glyma11g28160, Glyma20g23270, Glyma16g28640, Glyma14g22700, Glyma11g13370, Glyma13g28700, Glyma12g31700, Glyma02g06190, Glyma19g29100, Glyma05g37650, Glyma01g36850, Glyma09g41780, Glyma05g32790, Glyma06g07800, Glyma03g01090, Glyma09g39480, Glyma13g41410, Glyma06g17470, Glyma16g03030, Glyma17g01940, Glyma20g24150, Glyma12g02460, Glyma16g01680, Glyma17g03430, Glyma15g15100, Glyma08g08430, Glyma09g31470, Glyma03g36100, Glyma13g29220, Glyma08g45980, Glyma10g34390, Glyma17g04880, Glyma06g02910, Glyma08g12610, Glyma07g05170, Glyma10g34640, Glyma08g01440, Glyma01g39470, Glyma09g37720, Glyma06g32850, Glyma17g10510, Glyma18g04240, Glyma19g39150, Glyma13g05310, Glyma17g33900, Glyma12g03640, Glyma11g25480, Glyma10g05620, Glyma14g03830, Glyma14g06350, Glyma17g11210, Glyma17g02800, Glyma02g35350, Glyma20g10030, Glyma01g38470
