# Supplementary figures and images for: Transcript Profile of the Response of Two Soybean Genotypes to Potassium Deficiency
Source: PLoS One. 2012 Jul 5;7(7):e39856. doi: 10.1371/journal.pone.0039856 (PMC3390323; doi:10.1371/journal.pone.0039856)

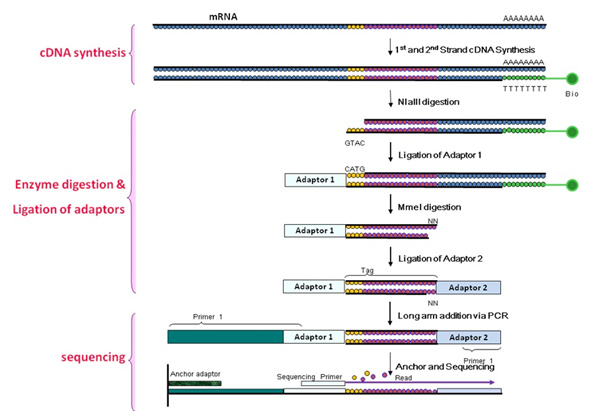


Supplemental Table S14. Principle and step of tag preparation

Supplement: Table S14 — Principle and step of tag preparation. (DOC) [file pone.0039856.s015.doc]
